# A new power G-family of distributions: Properties, estimation, and applications

**DOI:** 10.1371/journal.pone.0308094

**Published:** 2024-08-05

**Authors:** Ahmed M. Gemeay, Waleed Hamoud Alharbi, Alaa R. El-Alosey

**Affiliations:** Department of Mathematics, Faculty of Science, Tanta University, Tanta, Egypt; Cyprus International University Faculty of Engineering: Uluslararasi Kibris Universitesi Muhendislik Fakultesi, TÜRKIYE

## Abstract

This article suggests a new method to expand a family of life distributions by adding a parameter to the family, increasing its flexibility. It is called the extended Modi-G family of distributions. We derived the general statistical properties of the proposed family. Different methods of estimation were presented to estimate the parameters for the proposed family, such as maximum likelihood, ordinary least square, weighted least square, Anderson Darling, right-tailed Anderson-Darling, Cramér-von Mises, and maximum product of spacing methods. A special sub-model with three parameters called extended Modi exponential distribution was derived along with different shapes of its density and hazard functions. Randomly generated data sets and different estimation methods were used to illustrate the behavior of parameters of the proposal sub-model. To illustrate the importance of the proposed family over the other well-known methods, applications to medicine and geology data sets were analyzed.

## 1 Introduction

In probability distribution theory, choosing a particular probability distribution for modeling real-life phenomena could depend on whether the distribution is flexible. The tractability of a probability distribution might be helpful in theory because such distribution would be easy to work with, particularly with regard randomly generated data of different random samples. Still, the flexibility of probability distributions could be of interest to experts. It is preferable to use probability distributions that best fit the available data set rather than transform the existing one. Therefore, many attempts have been made lately to guarantee that the standard theoretical distributions are changed and developed. This could build their adaptability and increase their capacity to model real-life data sets.

Different methods could be put into use to expand the current standard distribution. For example, the flexibility of a distribution can be increased through generalization, which involves using the accessible generalized family of distributions. When a distribution is generalized, extra shape parameter(s) from the family of distributions utilized would have been added. The job of these additional shape parameter(s) is to change the tail weight of the resulting compound distribution, thereby inducing it with skewness. Generalizing classical distributions is an ancient practice, as important as many other practical problems in statistics. These generalizations introduced additional location, scale, or shape parameters to the original model. This branch of statistics has received considerable attention. Many general distribution classes have been derived in recent years. Azzalini [1] introduced the skew-normal distribution by adding an extra parameter to the normal distribution to increase flexibility. Mudholkar and Srivastava [2] proposed a technique for adding an extra parameter to a two-parameter Weibull distribution. Marshall and Olkin [3] introduced another method for adding a parameter to expand a family of distributions. Eugene et al. [4] introduced the beta generalized family of distributions. It was derived from the log-it of the beta random variable, and it has two extra shape parameters. Cordeiro and de Castro [5] created and studied another family of generalized distributions dependent on the Kumaraswamy distribution. Zografos and Balakrishnan [6] presented a gamma generalized family of distributions. Type II of gamma generalized family of distributions presented by Ristic and Balakrishnan [7]. The McDonald generalized family of distribution was generated from the McDonald random variables by Alexander et al. [8]. Alzaatreh et al. [9] presented a new method of generating families of continuous distributions called the T-X family. Mahdavi and Kundu [10] introduced a new method for deriving statistical distributions. For more surveys about methods of generating distributions, see Lee et al. [11], Jones [12], and Ahmad et al. [13].

A more adaptable distribution that works well in various circumstances is still required, even though several distributions in the literature may be used to evaluate data in many domains. The main aim of this paper is to propose a new family of generalized distributions. The family’s many mathematical properties are investigated, and several methods are used for parameter estimates. A new statistical model is derived using the exponential distribution as a baseline for our proposed family. The exponential distribution’s additional parameters changed the tail length and created uneven densities to the right and left. The behavior of new model estimators was examined via simulation, and all estimation methods showed the consistency property for all measures. Three real data sets were used to demonstrate the suggested model’s applicability compared to the other models.

The article is prepared as follows: The proposed family is presented in Section 2, and Section 3 demonstrates its statistical features, including the quantile function, moments, the moment generating function, incomplete moments, and the Rényi entropy. The maximum likelihood estimation (MLE), ordinary Least-Squares estimation (OLSE), weighted-least Squares estimation (WLSE), Anderson-Darling estimation (ADE), right-tailed Anderson-Darling estimation (RTADE), Cramér-von Mises estimation (CME) and maximum product of spacing (MPSE) techniques are described in Section 4 for estimating the proposed family parameters. Section 5 defines the extended Modi exponential distribution (ExMED). Estimating the ExMED parameters using simulation results in section 6. In Section 7, three real data sets illustrate the performance of the ExMED distribution. Finally, some final thoughts are offered in Section 8.

## 2 Family formulation

Modi et al. [14] presented a new Modi family of probability distributions. Their CDF and PDF are given by

$$\begin{array}{cc} F(x) & =\frac{\left(1+\alpha^{\beta }\right)G\left(x\right)}{\alpha^{\beta }+G\left(x\right)},\\ f(x) & =\frac{\alpha^{\beta }\left(1+\alpha^{\beta }\right)g\left(x\right)}{{\left[\alpha^{\beta }+G\left(x\right)\right]}^{2}}, \end{array}$$

respectively, where *x*, *α*, *β* > 0. They studied the statistical properties of Modi exponential distribution and applied them to two real data sets.

This section presents a relatively new family of generalized distributions called the extended Modi-G family of distributions. Its CDF is given by

$$\begin{array}{c} F\left(x;\alpha ,\theta ,\phi \right)=\frac{\left(1+\alpha \right)G{\left(x;\phi \right)}^{\theta }}{\alpha +G{\left(x;\phi \right)}^{\theta }}, \end{array}$$ (1)

where *x* ∈ *R*, *α*, *θ* > 0. Its PDF is defined as the following

$$\begin{array}{c} f\left(x;\alpha ,\theta ,\phi \right)=\frac{\theta \alpha \left(\alpha +1\right)G{\left(x;\phi \right)}^{\theta -1}g\left(x;\phi \right)}{{\left(\alpha +G{\left(x;\phi \right)}^{\theta }\right)}^{2}}, \end{array}$$ (2)

where *G*(*x*; *ϕ*) is the CDF of the baseline distribution.

Survival function (SF), hazard rate function (HRF), and reverse hazard rate function (RHRF) of the extended Modi-G family are given by (3)–(5), respectively.

$$\begin{array}{c} S\left(x;\alpha ,\theta ,\phi \right)=\frac{\alpha -\alpha G{\left(x;\phi \right)}^{\theta }}{\alpha +G{\left(x;\phi \right)}^{\theta }}, \end{array}$$ (3)

$$\begin{array}{c} h\left(x;\alpha ,\theta ,\phi \right)=\frac{\theta \alpha \left(\alpha +1\right)G{\left(x;\phi \right)}^{\theta -1}g\left(x;\phi \right)}{\left(\alpha -\alpha G{\left(x;\phi \right)}^{\theta }\right)\left(\alpha +G{\left(x;\phi \right)}^{\theta }\right)}, \end{array}$$ (4)

$$\begin{array}{c} r\left(x;\alpha ,\theta ,\phi \right)=\frac{\theta \alpha G{\left(x;\phi \right)}^{-1}g\left(x;\phi \right)}{\alpha +G{\left(x;\phi \right)}^{\theta }}. \end{array}$$ (5)

## 3 Mathematical properties

This section provides some mathematical properties of the extended Modi-G family of distributions, such as linear representation, quantile function, moments, moment-generating function, incomplete moments, and entropy.

### 3.1 Linear representation

A useful linear representation of CDF (1) and PDF (2) is introduced in this subsection. For −1 ≤ *x* ≤ 1, we have

$$\begin{array}{c} {\left(1+x\right)}^{-1}=\sum_{k=1}^{\infty }{\left(-1\right)}^{k-1}x^{k-1}, \end{array}$$ (6)

by applying the power series (6) in (1), we have

$$\begin{array}{c} F\left(x;\alpha ,\theta ,\phi \right)=\sum_{k=1}^{\infty }\frac{{\left(-1\right)}^{k-1}\left(\alpha +1\right)}{\alpha^{k}}G{\left(x;\phi \right)}^{\theta k},\;\;G{\left(x;\phi \right)}^{\theta }<\alpha , \end{array}$$ (7)

$$\begin{array}{c} f\left(x;\alpha ,\theta ,\phi \right)=\sum_{k=1}^{\infty }\frac{{\left(-1\right)}^{k-1}\left(\alpha +1\right)\theta k}{\alpha^{k}}G{\left(x;\phi \right)}^{\theta k-1}g\left(x;\phi \right). \end{array}$$ (8)

### 3.2 Quantile function

Let X be a random variable with CDF (1), then the quantile function (QF) of X is defined as the following

$$\begin{array}{c} Q\left(u\right)=F^{-1}\left(u\right)=G^{-1}{\left(\frac{\alpha -u+1}{\alpha u}\right)}^{-\frac{1}{\theta }}, \end{array}$$ (9)

where *G*^−1^ is the QF of the baseline distribution and *u* ∈ (0, 1). By setting *u* = 0.25, 0.5, 0.75, we have first, second, and third quartiles, respectively, which are used to determine Bowley’s skewness (BS) [15] as the following

$$\begin{array}{c} BS=\frac{Q(1/4)+Q(3/4)-2Q(1/2)}{Q(3/4)-Q(1/4)}, \end{array}$$ (10)

and Moor’s kurtosis (MK) [16] as the following

$$\begin{array}{c} MK=\frac{Q(7/8)-Q(5/8)+Q(3/8)-Q(1/8)}{Q(6/8)-Q(2/8)}. \end{array}$$ (11)

### 3.3 Moments and moment generating function

Moments play an important role in statistical analysis, especially in applications. The *r*^*th*^ moments of the extended Modi-G family of distribution are defined as the following

$$\begin{array}{c} \mu_{r}^{\prime }=E\left(X^{r}\right)=\int_{-\infty }^{\infty }x^{r}f\left(x;\alpha ,\theta ,\phi \right)dx, \end{array}$$ (12)

using (8) in (12), we have

$$\begin{array}{c} \mu_{r}^{\prime }=E\left(X^{r}\right)=\sum_{k=1}^{\infty }\frac{{\left(-1\right)}^{k-1}\left(\alpha +1\right)\theta k}{\alpha^{k}}\int_{-\infty }^{\infty }x^{r}G{\left(x;\phi \right)}^{\theta k-1}g\left(x;\phi \right)dx. \end{array}$$ (13)

The moment generating function (MGF) of extended Modi-G random variable X is given by

$$\begin{array}{c} M_{x}\left(t\right)=E\left(e^{tX}\right)=1+\sum_{m,k=1}^{\infty }\frac{{\left(-1\right)}^{k-1}\left(\alpha +1\right)\theta k}{\alpha^{k}}\frac{t^{m}}{m!}\int_{-\infty }^{\infty }x^{m}G{\left(x;\phi \right)}^{\theta k-1}g\left(x;\phi \right)dx, \end{array}$$ (14)

by replacing *t* by *it*, we have the characteristic function of the extended Modi-G family of distribution.

### 3.4 Incomplete moments

Let X be a random variable with PDF (2), then the *r*^*th*^ incomplete moments of it is given as the following

$$\begin{array}{cc} I_{r}\left(y;\alpha ,\theta ,\phi \right) & =\int_{-\infty }^{y}x^{r}f\left(x;\alpha ,\theta ,\phi \right)dx=\sum_{k=1}^{\infty }\frac{{\left(-1\right)}^{k-1}\left(\alpha +1\right)\theta k}{\alpha^{k}}\int_{-\infty }^{y}x^{r}G{\left(x;\phi \right)}^{\theta k-1}g\left(x;\phi \right)dx\\ & =\sum_{k=1}^{\infty }\frac{{\left(-1\right)}^{k-1}\left(\alpha +1\right)\theta k}{\alpha^{k}}\int_{0}^{G(y;\phi )}G^{-1}{\left(u\right)}^{r}u^{\theta k-1}du, \end{array}$$

The first incomplete moment of X is given by

$$\begin{array}{ccc} I_{1}\left(y;\alpha ,\theta ,\phi \right) & = & \sum_{k=1}^{\infty }\frac{{\left(-1\right)}^{k-1}\left(\alpha +1\right)\theta k}{\alpha^{k}}\int_{0}^{G(y;\phi )}G^{-1}\left(u\right)u^{\theta k-1}du. \end{array}$$ (15)

by using Eq (15), we have Lorenz, Bonferroni, and Zenga curves [17], respectively, as the following

$$\begin{array}{c} LN\left(p\right)=\frac{I_{1}\left(x_{p}\right)}{\mu },\;BN\left(p\right)=\frac{LN(p)}{p},\;ZN\left(p\right)=\frac{LN(p)-p}{p[1-LN(p)]}, \end{array}$$

where *F*(*x*_*p*_; *α*, *θ*, *ϕ*) = *p*.

Also, by using Eqs (1) and (15), we can determine the mean residual life (MRL) and the mean inactivity time (MIT), respectively, as the following

$$\begin{array}{c} MRL\left(t\right)=\frac{1-I_{1}\left(t\right)}{1-F(t)}-t,\;MIT\left(t\right)=t-\frac{I_{1}\left(t\right)}{F(t)}. \end{array}$$

### 3.5 Entropy

The entropy of a random variable X determines the randomness found in a probability distribution, and different types of entropies are not similarly useful for all applications.

Let *X* be a continuous random variable with, then Rényi entropy [18] is given by

$$\begin{array}{c} R_{r}\left(x;\alpha ,\theta ,\phi \right)=\frac{1}{1-r}\;\text{log}\;[\int_{-\infty }^{\infty }f{\left(x;\alpha ,\theta ,\phi \right)}^{r}dx],\;r>0,\;\;r\neq 1. \end{array}$$ (16)

From PDF (2), we have

$$\begin{array}{c} f{\left(x;\alpha ,\theta ,\phi \right)}^{r}=\frac{{\left(\theta \alpha \right)}^{r}{\left(\alpha +1\right)}^{r}G{\left(x;\phi \right)}^{r(\theta -1)}g{\left(x;\phi \right)}^{r}}{{\left(\alpha +G{\left(x;\phi \right)}^{\theta }\right)}^{2r}}=\frac{\theta^{r}{\left(\alpha +1\right)}^{r}G{\left(x;\phi \right)}^{r(\theta -1)}g{\left(x;\phi \right)}^{r}}{\alpha^{r}{\left(1+\frac{G{\left(x;\phi \right)}^{\theta }}{\alpha }\right)}^{2r}}, \end{array}$$

where

(1+x)-a=∑j=0∞(-aj)xj,(-aj)=(-1)j(a+j-1j),|x|<1,

then

f(x;α,θ,ϕ)r=θr(α+1)rG(x;ϕ)r(θ-1)g(x;ϕ)rαr∑j=0∞(-2rj)G(x;ϕ)jθαj=∑j=0∞(-2rj)θr(α+1)rG(x;ϕ)jθ+rθ-rg(x;ϕ)rαj+r. (17)

From Eq (17) in Eq (16), we have Rényi of the extended Modi-G family as the following

Rr(x;α,θ,ϕ)=11-rlog[∑j=0∞(-2rj)θr(α+1)rαj+r∫-∞∞G(x;ϕ)jθ+rθ-rg(x;ϕ)rdx]. (18)

Let X be a continuous random variable from the extended Modi-G family, then Tsallis entropy is given by

Hr(x;α,θ,ϕ)=11-r[∫-∞∞f(x;α,θ,ϕ)rdx-1],=11-r[∑j=0∞(-2rj)θr(α+1)rαj+r∫-∞∞G(x;ϕ)jθ+rθ-rg(x;ϕ)rdx-1]

Let X be a continuous random variable from the extended Modi-G family, then Shannon entropy [19] is given by

$$\begin{array}{cc} H(x;\alpha ,\theta ,\phi ) & =-E[\text{log}\;f(x;\alpha ,\theta ,\phi )]\\ & =-\text{log}\;[\frac{\theta (\alpha +1)}{\alpha }]-\left(\theta -1\right)\int_{-\infty }^{\infty }f\left(x;\alpha ,\theta ,\phi \right)\;\text{log}\;[G(x;\phi )]dx\\ & -\int_{-\infty }^{\infty }f\left(x;\alpha ,\theta ,\phi \right)\;\text{log}\;[g(x;\phi )]dx\\ & +2\sum_{k=1}^{\infty }\frac{{\left(-1\right)}^{k+1}}{k\alpha^{k}}\int_{-\infty }^{\infty }f\left(x;\alpha ,\theta ,\phi \right){\left[G(x;\phi )\right]}^{\theta \;k}dx. \end{array}$$

## 4 Estimation methods

In this section, we introduce a different method for estimating unknown parameters of the extended Modi-G family of distributions, such as maximum likelihood estimation (MLE), ordinary Least-Squares estimation (OLSE), weighted-Least Squares estimation (WLSE), Anderson-Darling estimation (ADE), right-tailed Anderson-Darling estimation (RTADE), Cramér-von Mises estimation (CME), and maximum product of spacing (MPSE).

### 4.1 Maximum likelihood estimation

It is the most common method used for estimating unknown parameters (for more details, see [20]). Let *X*_1_, *X*_2_,…*X*_*n*_ be a random sample with PDF (2), then the log-likelihood function is given by

$$\begin{array}{cc} L(x;\alpha ,\theta ,\phi ) & =n\;\text{log}\;[\theta \alpha (\alpha +1)]+\left(\theta -1\right)\sum_{i=1}^{n}\;\text{log}\;[G(x;\phi )]+\sum_{i=1}^{n}\;\text{log}\;[g(x;\phi )]\\ & -2\sum_{i=1}^{n}\;\text{log}\;[\alpha +G{\left(x;\phi \right)}^{\theta }]. \end{array}$$ (19)

By derivative Eq (19) to its parameters and equating the result equations to zero will provide us with the requested estimates. These derivatives are determined as follows

$$\begin{array}{cc} \frac{\partial }{\partial \alpha }L\left(x;\alpha ,\theta ,\phi \right) & =\frac{n(2\alpha +1)}{\alpha (\alpha +1)}-\sum_{i=1}^{n}\frac{2}{\alpha +G{\left(x;\phi \right)}^{\theta }}=0,\\ \frac{\partial }{\partial \theta }L\left(x;\alpha ,\theta ,\phi \right) & =\frac{n}{\theta }+\sum_{i=1}^{n}\;\text{log}\;[G(x;\phi )]-2\sum_{i=1}^{n}\frac{G{\left(x;\phi \right)}^{\theta }\;\text{log}\;[G(x;\phi )]}{\alpha +G{\left(x;\phi \right)}^{\theta }}=0,\\ \frac{\partial }{\partial \phi }L\left(x;\alpha ,\theta ,\phi \right) & =\left(\theta -1\right)\sum_{i=1}^{n}\frac{\frac{\partial }{\partial \phi }G\left(x_{i};\phi \right)}{G(x;\phi )}+\sum_{i=1}^{n}\frac{\frac{\partial }{\partial \phi }g\left(x_{i};\phi \right)}{g(x_{i};\phi )}\\ & -2\sum_{i=1}^{n}\frac{\theta G{\left(x;\phi \right)}^{\theta -1}\frac{\partial }{\partial \phi }G\left(x_{i};\phi \right)}{\alpha +G{\left(x;\phi \right)}^{\theta }}=0. \end{array}$$

The second derivative of each parameter is determined to construct the proposed model Hessian matrix as the following

$$\begin{array}{cc} \frac{\partial^{2}}{\partial \alpha^{2}}L\left(x;\alpha ,\theta ,\phi \right) & =-n\left[\frac{1}{\alpha^{2}}+\frac{1}{{\left(\alpha +1\right)}^{2}}\right]+\sum_{i=1}^{n}\frac{2}{{\left[\alpha +G{\left(x;\phi \right)}^{\theta }\right]}^{2}},\\ \frac{\partial^{2}}{\partial \alpha \partial \theta }L\left(x;\alpha ,\theta ,\phi \right) & =\sum_{i=1}^{n}\frac{2G{\left(x;\phi \right)}^{\theta }\;\text{log}\;\left[G\left(x;\phi \right)\right]}{{\left[\alpha +G{\left(x;\phi \right)}^{\theta }\right]}^{2}},\\ \frac{\partial^{2}}{\partial \alpha \partial \phi }L\left(x;\alpha ,\theta ,\phi \right) & =\sum_{i=1}^{n}\frac{2\theta G{\left(x;\phi \right)}^{\theta -1}\frac{\partial }{\partial \phi }G\left(x_{i};\phi \right)}{{\left[\alpha +G{\left(x;\phi \right)}^{\theta }\right]}^{2}},\\ \frac{\partial^{2}}{\partial \theta^{2}}L\left(x;\alpha ,\theta ,\phi \right) & =\frac{-n}{\theta^{2}}-2\sum_{i=1}^{n}\frac{\alpha G{\left(x;\phi \right)}^{\theta }\;{\text{log}}^{2}\;\left[G\left(x;\phi \right)\right]}{{\left[\alpha +G{\left(x;\phi \right)}^{\theta }\right]}^{2}},\\ \frac{\partial^{2}}{\partial \theta \partial \phi }L\left(x;\alpha ,\theta ,\phi \right) & =\sum_{i=1}^{n}\frac{\frac{\partial }{\partial \phi }G\left(x_{i};\phi \right)}{G(x;\phi )}-2\sum_{i=1}^{n}\frac{G{\left(x;\phi \right)}^{\theta -1}\frac{\partial }{\partial \phi }G\left(x_{i};\phi \right)}{{\left[\alpha +G{\left(x;\phi \right)}^{\theta }\right]}^{2}}\\ & (\left[\alpha +G{\left(x;\phi \right)}^{\theta }\right]\left\{1+\theta \;\text{log}\;\left[G\left(x;\phi \right)\right]\right\}-\theta G{\left(x;\phi \right)}^{\theta }\;\text{log}\;\left[G\left(x;\phi \right)\right]),\\ \frac{\partial^{2}}{\partial \phi^{2}}L\left(x;\alpha ,\theta ,\phi \right) & =\left(\theta -1\right)\sum_{i=1}^{n}\frac{G\left(x;\phi \right)\frac{\partial^{2}}{\partial \phi^{2}}G\left(x_{i};\phi \right)-{\left[\frac{\partial }{\partial \phi }G\left(x_{i};\phi \right)\right]}^{2}}{G{\left(x;\phi \right)}^{2}}\\ & +\sum_{i=1}^{n}\frac{g\left(x_{i};\phi \right)\frac{\partial^{2}}{\partial \phi^{2}}g\left(x_{i};\phi \right)-{\left[\frac{\partial }{\partial \phi }g\left(x_{i};\phi \right)\right]}^{2}}{g{\left(x_{i};\phi \right)}^{2}}-2\theta \sum_{i=1}^{n}\frac{G{\left(x;\phi \right)}^{\theta -1}}{{\left[\alpha +G{\left(x;\phi \right)}^{\theta }\right]}^{2}}\\ & \left(\left[\alpha +G{\left(x;\phi \right)}^{\theta }\right]\{\frac{\partial^{2}}{\partial \phi^{2}}G\left(x_{i};\phi \right)+\left(\theta -1\right)G{\left(x;\phi \right)}^{-1}{\left[\frac{\partial }{\partial \phi }G\left(x_{i};\phi \right)\right]}^{2}\right\}\\ & -\theta G{\left(x;\phi \right)}^{\theta -1}{\left[\frac{\partial }{\partial \phi }G\left(x_{i};\phi \right)\right]}^{2}). \end{array}$$

Now, after determining the inverse of the Hessian matrix, we obtained the covariance matrix of our estimators. By calculating its diagonal square root, we obtained the standard errors of our estimators.

### 4.2 Ordinary Least-Squares and Weighted-Least Squares estimation

Let *x*_1:*n*_, *x*_2:*n*_, …, *x*_*n*:*n*_ be the order statistics of a random sample of size *n* from the extended Modi-G family of distributions, where

$$\begin{array}{c} OL\left(x_{i:n};\alpha ,\theta ,\phi \right)=\sum_{i=1}^{n}{\left[\frac{\left(1+\alpha \right)G{\left(x_{i:n};\phi \right)}^{\theta }}{\alpha +G{\left(x_{i:n};\phi \right)}^{\theta }}-\frac{i}{n+1}\right]}^{2}, \end{array}$$ (20)

estimates can be obtained by solving simultaneously the three non-linear equations obtained from minimizing Eq (20) to its parameters (for further detail, see [21]).

Similarly, WLSE is determined by minimizing the following equation

$$\begin{array}{c} W\left(x_{i:n};\alpha ,\theta ,\phi \right)=\sum_{i=1}^{n}\frac{{\left(n+1\right)}^{2}\left(n+2\right)}{i(n-i+1)}{\left[\frac{(1+\alpha G{\left(x_{i:n};\phi \right)}^{\theta }}{\alpha +G{\left(x_{i:n};\phi \right)}^{\theta }}-\frac{i}{n+1}\right]}^{2}. \end{array}$$

### 4.3 Anderson-Darling and right-tail Anderson-Darling estimation

The ADE of unknown parameters of the extended Modi-G family of distributions are obtained by minimizing the following equation

$$\begin{array}{c} AD(x_{i:n};\alpha ,\theta ,\phi )=-n-\frac{1}{n}\sum_{i=1}^{n}\left(2i-1\right)[\text{log}\;\frac{\left(1+\alpha \right)G{\left(x_{i:n};\phi \right)}^{\theta }}{\alpha +G{\left(x_{i:n};\phi \right)}^{\theta }}+\text{log}\;\frac{\alpha -\alpha G{\left(x_{i:n};\phi \right)}^{\theta }}{\alpha +G{\left(x_{i:n};\phi \right)}^{\theta }}]. \end{array}$$

Similarly, the RTADE of parameters can be calculated by minimizing the following equation

$$\begin{array}{c} R=\frac{n}{2}-2\sum_{i=1}^{n}\;\text{log}\;\frac{\left(1+\alpha \right)G{\left(x_{i:n};\phi \right)}^{\theta }}{\alpha +G{\left(x_{i:n};\phi \right)}^{\theta }}-\frac{1}{n}\sum_{i=1}^{n}\left(2i-1\right)\;\text{log}\;\frac{\alpha -\alpha G{\left(x_{i:n};\phi \right)}^{\theta }}{\alpha +G{\left(x_{i:n};\phi \right)}^{\theta }}. \end{array}$$

### 4.4 Cramér-von Mises estimation

The CVME of unknown parameters of the extended Modi-G family of distributions are obtained by minimizing the following equation(for more details, see [22])

$$\begin{array}{c} CM\left(x_{i:n};\alpha ,\theta ,\phi \right)=\frac{1}{12n}+\sum_{i=1}^{n}{\left[\frac{\left(1+\alpha \right)G{\left(x_{i:n};\phi \right)}^{\theta }}{\alpha +G{\left(x_{i:n};\phi \right)}^{\theta }}-\frac{2i-1}{2n}\right]}^{2}. \end{array}$$

### 4.5 Maximum product of spacings estimation

The maximum product of spacings (MPSE) [23] method is used to estimate the parameters of continuous univariate models as an alternative to the ML method. The uniform spacings of a random sample of size n from the extended Modi-G family can be defined by

$$\begin{array}{c} D_{i}=\frac{\left(1+\alpha \right)G{\left(x_{i:n};\phi \right)}^{\theta }}{\alpha +G{\left(x_{i:n};\phi \right)}^{\theta }}-\frac{\left(1+\alpha \right)G{\left(x_{i-1:n};\phi \right)}^{\theta }}{\alpha +G{\left(x_{i-1:n};\phi \right)}^{\theta }}. \end{array}$$

where *D*_*i*_ denotes to the uniform spacings, *F*(*x*_0_) = 0, *F*(*x*_*n*+1_ = 1) and $\sum_{i=1}^{n+1}D_{i}=1$. MPS estimators of parameters can be obtained by maximizing

$$\begin{array}{c} G=\frac{1}{n+1}\sum_{i=1}^{n+1}\;\text{log}\;\left(D_{i}\right). \end{array}$$

## 5 A special sub-model

In this section, we defined a two-parameter sub-model of the proposed family by taking the CDF of the baseline distribution following the exponential distribution, which is called extended Modi exponential distribution (ExMED). Then, CDF and PDF of ExMED are given, respectively, by

$$\begin{array}{c} F\left(x\right)=\frac{\left(\alpha +1\right){\left(1-e^{-bx}\right)}^{\theta }}{\alpha +{\left(1-e^{-bx}\right)}^{\theta }}, \end{array}$$ (21)

$$\begin{array}{c} f\left(x\right)=\frac{\alpha \left(\alpha +1\right)b\theta {\left(1-e^{-bx}\right)}^{\theta }}{(e^{bx}-1){\left(\alpha +{\left(1-e^{-bx}\right)}^{\theta }\right)}^{2}}. \end{array}$$ (22)

Its SF, HRZ, and RHRF are, respectively, given by the following relation

$$\begin{array}{c} S=\alpha (\frac{\alpha +1}{\alpha +{\left(1-e^{-bx}\right)}^{\theta }}-1), \end{array}$$ (23)

$$\begin{array}{c} h=\frac{\left(\alpha +1\right)b\theta {\left(1-e^{-bx}\right)}^{\theta }}{\left(1-e^{bx})({\left(1-e^{-bx}\right)}^{\theta }-1)(\alpha +{\left(1-e^{-bx}\right)}^{\theta }\right)}, \end{array}$$ (24)

$$\begin{array}{c} r=\frac{\alpha b\theta }{\left(e^{bx}-1)(\alpha +{\left(1-e^{-bx}\right)}^{\theta }\right)}. \end{array}$$ (25)

**Fig 1:**
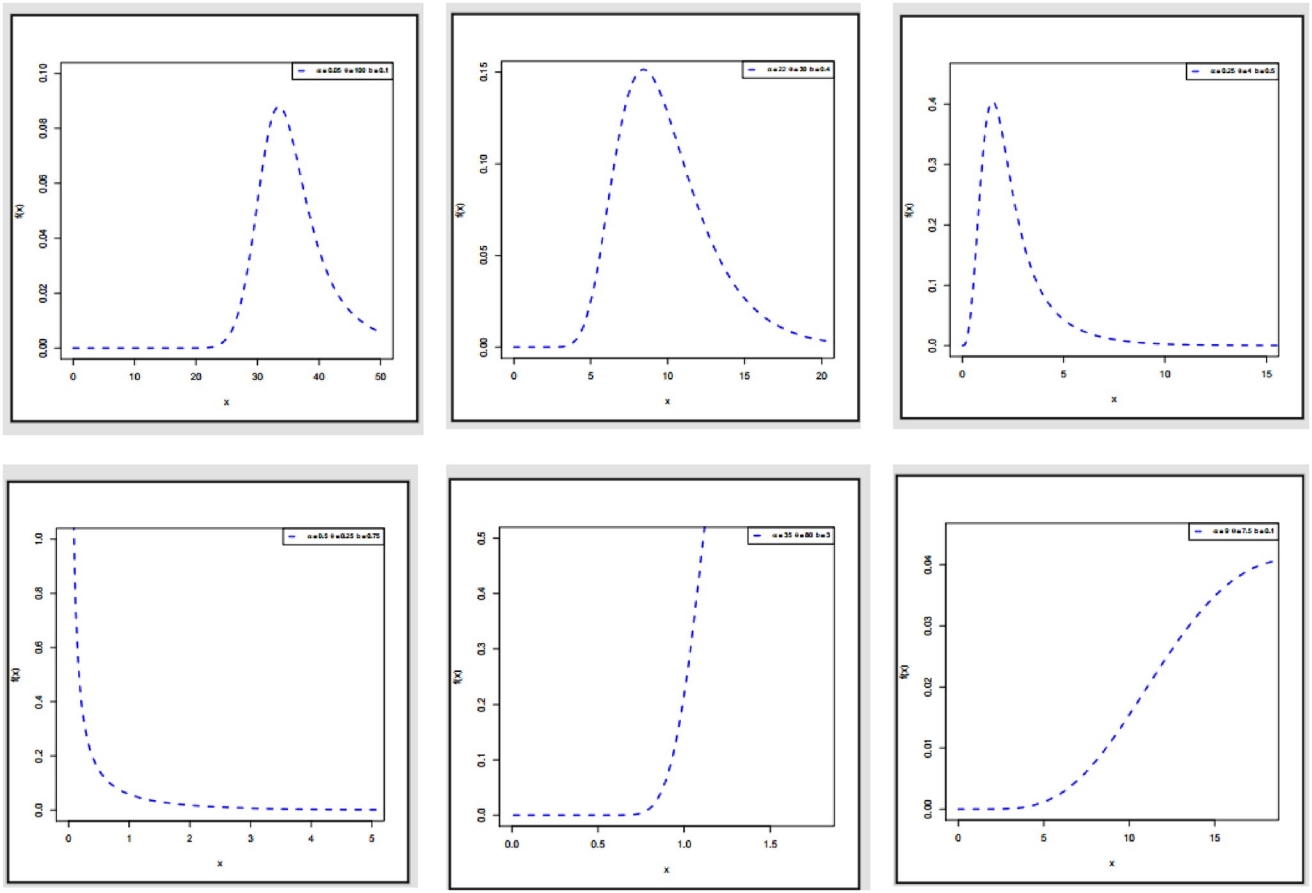
Plots of PDF of ExMED with various parameters values.

**Fig 2:**
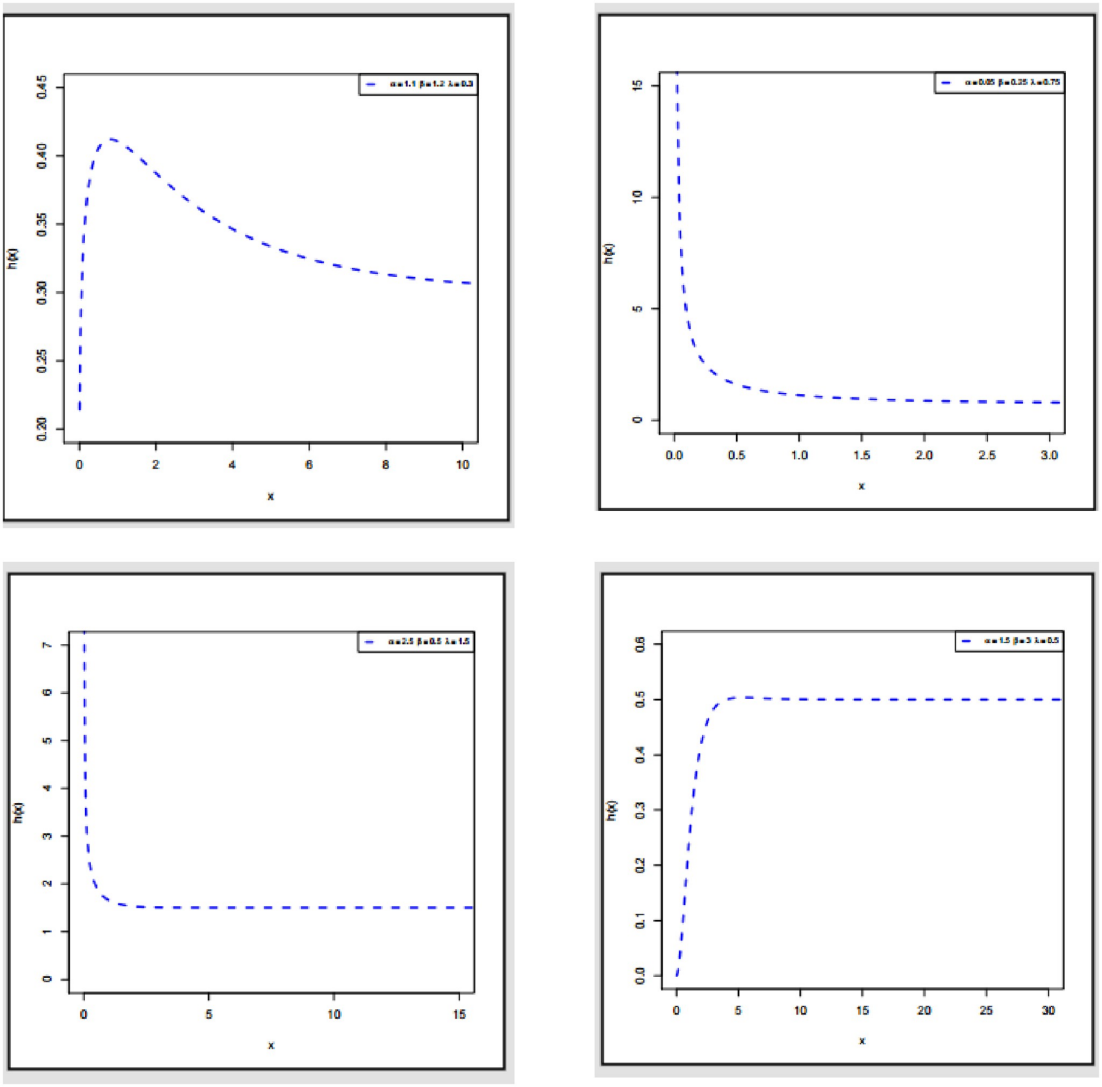
Plots of HRF of ExMED with various parameters values.

Figs 1 and 2 display the PDF and HRF plots of ExMED, respectively. As these figures demonstrate, the ExMED can handle decreasing, decreasing-constant, increasing, and increasing constant hazard rate functions. In addition, some densities are symmetrical, left-skewed, right-skewed, J-shaped, and reversed-J-shaped.

The quantile function of ExMED is given by

$$\begin{array}{c} Q\left(u\right)=\frac{1}{b}\;\text{log}\;[\frac{1}{1-{\left(\frac{\alpha u}{\alpha -p+1}\right)}^{1/\theta }}]. \end{array}$$ (26)

Let *u* ∼ uniform (0, 1). Then, using the ExMED’s QF, one may use the formula to produce random data sets of size *n* from this distribution.

$$\begin{array}{c} x_{i}=\frac{1}{b}\;\text{log}\;[\frac{1}{1-{\left(\frac{\alpha u_{i}}{\alpha -p+1}\right)}^{1/\theta }}],\;i=1,2,\ldots ,n. \end{array}$$ (27)

**Fig 3:**
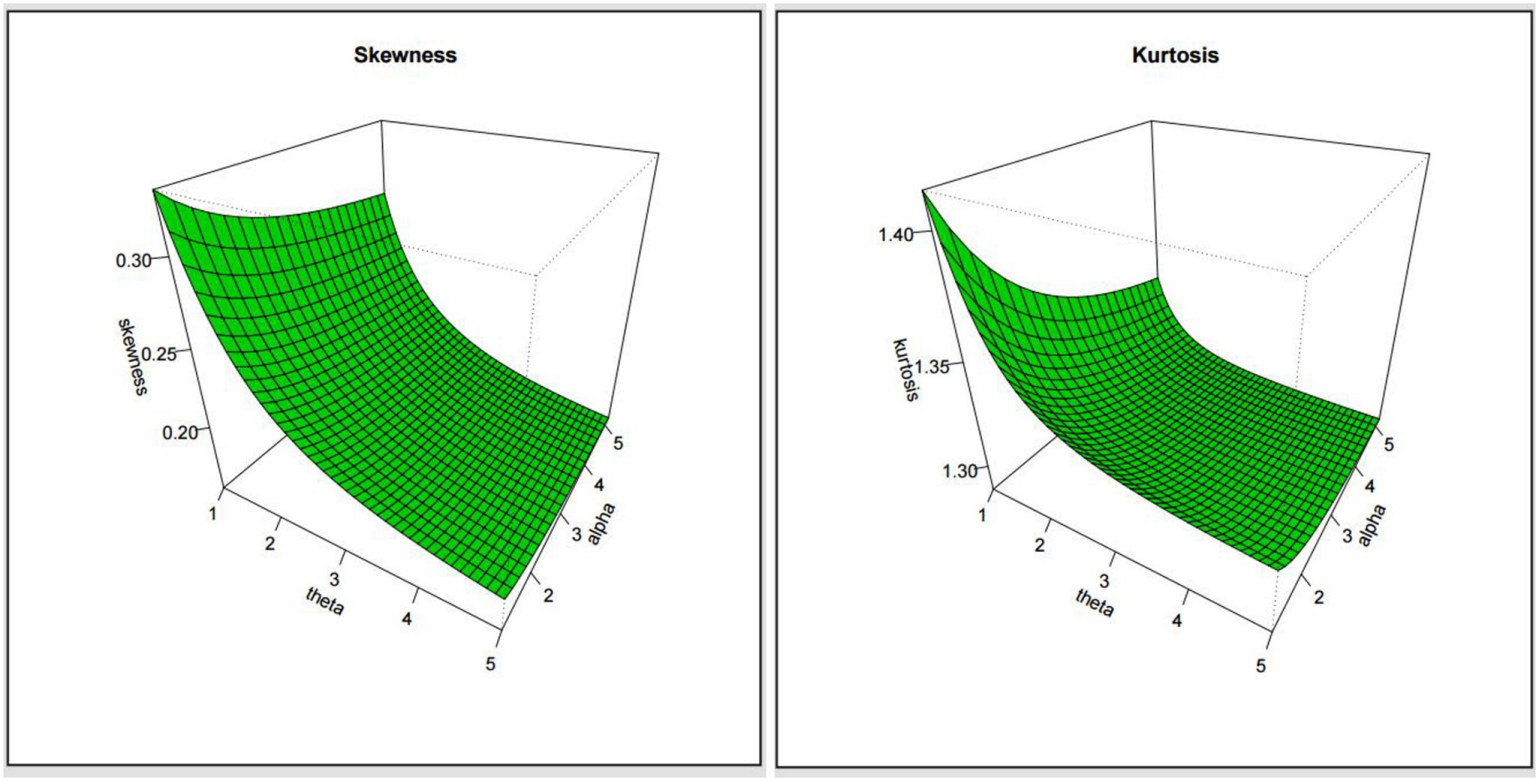
Plots of skewness and kurtosis of the ExMED model.

By Eqs (10), (11) and (26), we can determine BS and MK for ExMED, respectively. Fig 3 shows the skewness and kurtosis plots of the ExMED model for *b* = 2, along with several parametric values of *α* and *θ*.

## 6 Simulation results of estimation methods

We explore the performance of the aforementioned estimation methods in estimating the ExME parameters using simulation results. We consider various sample sizes, *n* = 20, 70, 100, 250, 1000, and various parametric values. We generate *n* = 1000 random samples from the ExMED and determine the average estimates (AESTs), the average absolute biases (ABs), average mean square error (AMSEs), and average mean relative estimates (AMREs) for all sample sizes and parameter combinations using the R software©.

The following respective equations can calculate the AESTs, ABs, AMSEs, and AMREs:

$$\begin{array}{cc} AESTs & =\frac{1}{N}\sum_{i=1}^{N}\hat{\theta },\;\;\;\;ABs=\frac{1}{N}\sum_{i=1}^{N}\left|\hat{\theta }-\theta \right|,\;\;\;\;AMSEs=\frac{1}{N}\sum_{i=1}^{N}{\left(\hat{\theta }-\theta \right)}^{2},\;\;\;\;\\ AMREs & =\frac{1}{N}\sum_{i=1}^{N}\left|\hat{\theta }-\theta \right|/\theta , \end{array}$$

where ***θ*** = (*α*, *θ*, *b*)′.

The results of the simulation study, including AESTs, ABs, AMSEs, and AMREs, were reported in Tables 1–4. The row indicating ∑ *Ranks* gives the partial sum of the ranks of ABs, AMSEs, and AMREs. A superscript indicates the rank of each of the estimators among all the estimators for that metric.

**Table 1:** Simulation results of the AESTs, ABs, AMSEs, and AMREs of ExMED for (*α* = 1.5, *θ* = 0.5, *b* = 0.75).

n	Est.	Est. Par.	MLE	ADE	CVME	MPSE	LSE	RTADE	WLSE
20	AESTs	$\hat{\alpha }$	1.02598	1.0436	1.01467	1.04455	1.00758	1.0259	1.04117
		$\hat{\theta }$	0.56327	0.55904	0.55069	0.54572	0.55519	0.55548	0.54407
		$\hat{b}$	0.72659	0.71025	0.68793	0.68653	0.6975	0.70364	0.68926
	ABs	$\hat{\alpha }$	0.66285^{4}^	0.67017^{6}^	0.6868^{7}^	0.5987^{1}^	0.66785^{5}^	0.65808^{2}^	0.66077^{3}^
		$\hat{\theta }$	0.10638^{4}^	0.10785^{7}^	0.09926^{1}^	0.10304^{3}^	0.10658^{5}^	0.10697^{6}^	0.10078^{2}^
		$\hat{b}$	0.2111^{1}^	0.21783^{4}^	0.21303^{2}^	0.22697^{7}^	0.2238^{5}^	0.21355^{3}^	0.22575^{6}^
	AMSEs	$\hat{\alpha }$	0.54737^{4}^	0.5499^{5}^	0.5845^{7}^	0.50899^{1}^	0.55601^{6}^	0.54165^{3}^	0.54007^{2}^
		$\hat{\theta }$	0.0182^{5}^	0.01895^{7}^	0.01644^{1}^	0.01697^{3}^	0.01816^{4}^	0.01853^{6}^	0.01661^{2}^
		$\hat{b}$	0.06387^{2}^	0.06712^{4}^	0.06382^{1}^	0.07291^{7}^	0.0706^{5}^	0.06474^{3}^	0.07185^{6}^
	AMREs	$\hat{\alpha }$	0.4419^{4}^	0.44678^{6}^	0.45786^{7}^	0.39913^{1}^	0.44523^{5}^	0.43872^{2}^	0.44051^{3}^
		$\hat{\theta }$	0.21276^{4}^	0.21571^{7}^	0.19853^{1}^	0.20608^{3}^	0.21315^{5}^	0.21393^{6}^	0.20156^{2}^
		$\hat{b}$	0.28147^{1}^	0.29045^{4}^	0.28404^{2}^	0.30262^{7}^	0.2984^{5}^	0.28473^{3}^	0.301^{6}^
	∑ *Ranks*		29^{1.5}^	50^{7}^	29^{1.5}^	33^{4}^	45^{6}^	34^{5}^	32^{3}^
70	AESTs	$\hat{\alpha }$	1.14127	1.12497	1.0863	1.10967	1.0908	1.09893	1.06346
		$\hat{\theta }$	0.54775	0.54197	0.5451	0.53295	0.54142	0.54687	0.54588
		$\hat{b}$	0.73515	0.70553	0.68365	0.67774	0.69025	0.71426	0.69224
	ABs	$\hat{\alpha }$	0.62419^{3}^	0.62816^{4}^	0.63649^{7}^	0.49656^{1}^	0.63058^{5}^	0.62043^{2}^	0.63277^{6}^
		$\hat{\theta }$	0.07169^{3}^	0.07035^{2}^	0.0727^{4}^	0.06844^{1}^	0.07299^{6}^	0.07458^{7}^	0.07285^{5}^
		$\hat{b}$	0.148^{1}^	0.15741^{3}^	0.16092^{6}^	0.15928^{4}^	0.16061^{5}^	0.15625^{2}^	0.16118^{7}^
	AMSEs	$\hat{\alpha }$	0.48851^{2}^	0.49622^{4}^	0.51288^{7}^	0.41607^{1}^	0.50769^{5}^	0.49315^{3}^	0.50874^{6}^
		$\hat{\theta }$	0.00837^{3}^	0.0079^{1}^	0.00908^{6}^	0.00805^{2}^	0.00887^{5}^	0.0094^{7}^	0.00865^{4}^
		$\hat{b}$	0.03397^{1}^	0.03664^{3}^	0.03702^{5}^	0.0361^{2}^	0.03835^{7}^	0.03668^{4}^	0.03795^{6}^
	AMREs	$\hat{\alpha }$	0.41612^{3}^	0.41877^{4}^	0.42432^{7}^	0.33104^{1}^	0.42039^{5}^	0.41362^{2}^	0.42184^{6}^
		$\hat{\theta }$	0.14337^{3}^	0.14071^{2}^	0.14539^{4}^	0.13687^{1}^	0.14598^{6}^	0.14916^{7}^	0.1457^{5}^
		$\hat{b}$	0.19734^{1}^	0.20987^{3}^	0.21456^{6}^	0.21238^{4}^	0.21415^{5}^	0.20834^{2}^	0.21491^{7}^
	∑ *Ranks*		20^{2}^	26^{3}^	52^{6.5}^	17^{1}^	49^{5}^	36^{4}^	52^{6.5}^
100	AESTs	$\hat{\alpha }$	1.15095	1.13694	1.14011	1.17088	1.09915	1.1167	1.13889
		$\hat{\theta }$	0.54244	0.54145	0.54027	0.52237	0.53465	0.5461	0.53589
		$\hat{b}$	0.71652	0.70362	0.70555	0.67851	0.67875	0.71084	0.69417
	ABs	$\hat{\alpha }$	0.59635^{2}^	0.62487^{7}^	0.60619^{4}^	0.44334^{1}^	0.61894^{6}^	0.61391^{5}^	0.6024^{3}^
		$\hat{\theta }$	0.06372^{5}^	0.06295^{4}^	0.06413^{6}^	0.05405^{1}^	0.06127^{3}^	0.06852^{7}^	0.06089^{2}^
		$\hat{b}$	0.12933^{1}^	0.14306^{5}^	0.14535^{7}^	0.13907^{4}^	0.14521^{6}^	0.13394^{2}^	0.13791^{3}^
	AMSEs	$\hat{\alpha }$	0.45927^{2}^	0.49157^{7}^	0.47456^{4}^	0.36844^{1}^	0.49081^{6}^	0.48176^{5}^	0.46597^{3}^
		$\hat{\theta }$	0.00696^{5}^	0.00647^{4}^	0.00716^{6}^	0.00504^{1}^	0.00631^{3}^	0.00779^{7}^	0.0063^{2}^
		$\hat{b}$	0.02508^{1}^	0.03026^{5}^	0.03207^{7}^	0.02827^{4}^	0.03134^{6}^	0.02742^{2}^	0.02815^{3}^
	AMREs	$\hat{\alpha }$	0.39756^{2}^	0.41658^{7}^	0.40413^{4}^	0.29556^{1}^	0.41263^{6}^	0.40927^{5}^	0.4016^{3}^
		$\hat{\theta }$	0.12743^{5}^	0.1259^{4}^	0.12827^{6}^	0.1081^{1}^	0.12254^{3}^	0.13705^{7}^	0.12179^{2}^
		$\hat{b}$	0.17244^{1}^	0.19074^{5}^	0.19381^{7}^	0.18543^{4}^	0.19362^{6}^	0.17859^{2}^	0.18387^{3}^
	∑ *Ranks*		24^{2.5}^	48^{6}^	51^{7}^	18^{1}^	45^{5}^	42^{4}^	24^{2.5}^
250	AESTs	$\hat{\alpha }$	1.26167	1.23313	1.22882	1.3002	1.2083	1.22848	1.25177
		$\hat{\theta }$	0.52521	0.52567	0.52781	0.51145	0.52667	0.52967	0.52388
		$\hat{b}$	0.72076	0.7125	0.70394	0.70192	0.69855	0.71139	0.70387
	ABs	$\hat{\alpha }$	0.51145^{2}^	0.52001^{3}^	0.55749^{7}^	0.26758^{1}^	0.55151^{6}^	0.54019^{5}^	0.52945^{4}^
		$\hat{\theta }$	0.04184^{2}^	0.04378^{3}^	0.04467^{5}^	0.03662^{1}^	0.04641^{6}^	0.0483^{7}^	0.04461^{4}^
		$\hat{b}$	0.0894^{1}^	0.0991^{4}^	0.10755^{6}^	0.0903^{2}^	0.11192^{7}^	0.10154^{5}^	0.09849^{3}^
	AMSEs	$\hat{\alpha }$	0.35116^{2}^	0.36275^{3}^	0.40135^{6}^	0.20913^{1}^	0.40173^{7}^	0.39027^{5}^	0.37738^{4}^
		$\hat{\theta }$	0.0029^{2}^	0.00315^{3}^	0.0033^{5}^	0.00233^{1}^	0.00349^{6}^	0.00386^{7}^	0.00325^{4}^
		$\hat{b}$	0.01263^{2}^	0.01477^{3}^	0.01782^{6}^	0.01241^{1}^	0.01905^{7}^	0.01583^{5}^	0.0148^{4}^
	AMREs	$\hat{\alpha }$	0.34097^{2}^	0.34667^{3}^	0.37166^{7}^	0.17839^{1}^	0.36767^{6}^	0.36012^{5}^	0.35297^{4}^
		$\hat{\theta }$	0.08368^{2}^	0.08756^{3}^	0.08933^{5}^	0.07323^{1}^	0.09281^{6}^	0.0966^{7}^	0.08922^{4}^
		$\hat{b}$	0.1192^{1}^	0.13213^{4}^	0.1434^{6}^	0.1204^{2}^	0.14923^{7}^	0.13539^{5}^	0.13131^{3}^
	∑ *Ranks*		16^{2}^	29^{3}^	53^{6}^	11^{1}^	58^{7}^	51^{5}^	34^{4}^
1000	AESTs	$\hat{\alpha }$	1.48331	1.49639	1.40253	1.473	1.43846	1.40196	1.47527
		$\hat{\theta }$	0.50664	0.506	0.5111	0.49945	0.50844	0.51325	0.50785
		$\hat{b}$	0.74301	0.74116	0.72609	0.73808	0.72828	0.73302	0.74018
	ABs	$\hat{\alpha }$	0.38076^{2}^	0.40507^{4}^	0.43195^{6}^	0.04727^{1}^	0.43332^{7}^	0.41057^{5}^	0.39191^{3}^
		$\hat{\theta }$	0.02241^{3}^	0.02306^{4}^	0.0255^{6}^	0.01463^{1}^	0.02508^{5}^	0.0286^{7}^	0.02214^{2}^
		$\hat{b}$	0.04868^{2}^	0.05374^{3}^	0.06652^{7}^	0.03992^{1}^	0.06518^{6}^	0.05387^{4}^	0.05557^{5}^
	AMSEs	$\hat{\alpha }$	0.21268^{2}^	0.23696^{5}^	0.2573^{6}^	0.02838^{1}^	0.26673^{7}^	0.23596^{4}^	0.22061^{3}^
		$\hat{\theta }$	0.00079^{2}^	0.00084^{4}^	0.00106^{6}^	0.00038^{1}^	0.00101^{5}^	0.00135^{7}^	0.00081^{3}^
		$\hat{b}$	0.00373^{2}^	0.00447^{3}^	0.00686^{6}^	0.00253^{1}^	0.00692^{7}^	0.00457^{4}^	0.00471^{5}^
	AMREs	$\hat{\alpha }$	0.25384^{2}^	0.27005^{4}^	0.28797^{6}^	0.03151^{1}^	0.28888^{7}^	0.27371^{5}^	0.26127^{3}^
		$\hat{\theta }$	0.04482^{3}^	0.04611^{4}^	0.05101^{6}^	0.02927^{1}^	0.05016^{5}^	0.0572^{7}^	0.04427^{2}^
		$\hat{b}$	0.06491^{2}^	0.07165^{3}^	0.08869^{7}^	0.05322^{1}^	0.08691^{6}^	0.07183^{4}^	0.07409^{5}^
	∑ *Ranks*		20^{2}^	34^{4}^	56^{7}^	9^{1}^	55^{6}^	47^{5}^	31^{3}^

**Table 2:** Simulation results of the AESTs, ABs, AMSEs, and AMREs of ExMED for (*α* = 2, *θ* = 1.5, *b* = 3).

n	Est.	Est. Par.	MLE	ADE	CVME	MPSE	LSE	RTADE	WLSE
20	AESTs	$\hat{\alpha }$	0.99942	1.00161	1.06546	1.24657	1.04347	0.99147	1.01094
		$\hat{\theta }$	1.7671	1.70786	1.72729	1.60622	1.69915	1.73393	1.69311
		$\hat{b}$	2.71496	2.63595	2.71346	2.58455	2.61225	2.63507	2.61707
	ABs	$\hat{\alpha }$	1.19554^{6}^	1.17489^{4}^	1.09302^{2}^	0.88648^{1}^	1.12482^{3}^	1.18054^{5}^	1.20001^{7}^
		$\hat{\theta }$	0.39408^{5}^	0.37592^{3}^	0.404^{7}^	0.37253^{1}^	0.39751^{6}^	0.38861^{4}^	0.37471^{2}^
		$\hat{b}$	0.72256^{1}^	0.75249^{2}^	0.7689^{4}^	0.80276^{6}^	0.80037^{5}^	0.75933^{3}^	0.80653^{7}^
	AMSEs	$\hat{\alpha }$	1.67527^{5}^	1.64332^{4}^	1.54134^{2}^	1.26581^{1}^	1.5828^{3}^	1.68528^{6}^	1.69297^{7}^
		$\hat{\theta }$	0.25405^{4}^	0.23887^{3}^	0.27256^{7}^	0.22026^{1}^	0.2585^{6}^	0.25419^{5}^	0.22868^{2}^
		$\hat{b}$	0.75539^{1}^	0.80646^{2}^	0.86433^{4}^	0.91608^{5}^	0.93406^{7}^	0.82688^{3}^	0.92997^{6}^
	AMREs	$\hat{\alpha }$	0.59777^{6}^	0.58744^{4}^	0.54651^{2}^	0.44324^{1}^	0.56241^{3}^	0.59027^{5}^	0.60001^{7}^
		$\hat{\theta }$	0.26272^{5}^	0.25061^{3}^	0.26933^{7}^	0.24836^{1}^	0.265^{6}^	0.25908^{4}^	0.24981^{2}^
		$\hat{b}$	0.24085^{1}^	0.25083^{2}^	0.2563^{4}^	0.26759^{6}^	0.26679^{5}^	0.25311^{3}^	0.26884^{7}^
	∑ *Ranks*		34^{3}^	27^{2}^	39^{5}^	23^{1}^	44^{6}^	38^{4}^	47^{7}^
70	AESTs	$\hat{\alpha }$	1.39598	1.3903	1.36722	1.75431	1.38325	1.35952	1.3957
		$\hat{\theta }$	1.61348	1.59274	1.61233	1.51491	1.60743	1.63532	1.58493
		$\hat{b}$	2.85767	2.82121	2.84508	2.85786	2.83888	2.84762	2.80967
	ABs	$\hat{\alpha }$	0.8699^{3}^	0.89498^{7}^	0.8905^{5}^	0.41394^{1}^	0.86596^{2}^	0.891^{6}^	0.874^{4}^
		$\hat{\theta }$	0.21917^{4}^	0.21519^{3}^	0.22498^{6}^	0.21329^{2}^	0.22012^{5}^	0.23406^{7}^	0.20675^{1}^
		$\hat{b}$	0.42278^{2}^	0.43074^{3}^	0.45061^{7}^	0.41967^{1}^	0.44977^{6}^	0.441^{4}^	0.44629^{5}^
	AMSEs	$\hat{\alpha }$	0.95602^{4}^	0.99463^{7}^	0.98498^{6}^	0.42143^{1}^	0.9533^{3}^	0.98223^{5}^	0.94223^{2}^
		$\hat{\theta }$	0.08061^{4}^	0.07905^{3}^	0.08407^{6}^	0.07191^{1}^	0.08247^{5}^	0.09395^{7}^	0.07397^{2}^
		$\hat{b}$	0.27233^{2}^	0.27965^{3}^	0.30645^{7}^	0.26149^{1}^	0.29808^{6}^	0.29507^{5}^	0.29005^{4}^
	AMREs	$\hat{\alpha }$	0.43495^{3}^	0.44749^{7}^	0.44525^{5}^	0.20697^{1}^	0.43298^{2}^	0.4455^{6}^	0.437^{4}^
		$\hat{\theta }$	0.14611^{4}^	0.14346^{3}^	0.14999^{6}^	0.14219^{2}^	0.14675^{5}^	0.15604^{7}^	0.13783^{1}^
		$\hat{b}$	0.14093^{2}^	0.14358^{3}^	0.1502^{7}^	0.13989^{1}^	0.14992^{6}^	0.147^{4}^	0.14876^{5}^
	∑ *Ranks*		28^{2.5}^	39^{4}^	55^{7}^	11^{1}^	40^{5}^	51^{6}^	28^{2.5}^
100	AESTs	$\hat{\alpha }$	1.51812	1.57183	1.55298	1.8303	1.57519	1.55684	1.55489
		$\hat{\theta }$	1.58801	1.57129	1.56893	1.48439	1.55608	1.56157	1.57004
		$\hat{b}$	2.93101	2.92052	2.91211	2.87831	2.91207	2.90345	2.92196
	ABs	$\hat{\alpha }$	0.67894^{7}^	0.63343^{2}^	0.64223^{3}^	0.28037^{1}^	0.65028^{4}^	0.65824^{6}^	0.65607^{5}^
		$\hat{\theta }$	0.17957^{7}^	0.17159^{5}^	0.17816^{6}^	0.15713^{1}^	0.16867^{3}^	0.16754^{2}^	0.17123^{4}^
		$\hat{b}$	0.34064^{4}^	0.33035^{2}^	0.33987^{3}^	0.31944^{1}^	0.35463^{7}^	0.3414^{5}^	0.34957^{6}^
	AMSEs	$\hat{\alpha }$	0.58391^{7}^	0.51874^{2}^	0.5388^{3}^	0.2144^{1}^	0.54765^{5}^	0.54469^{4}^	0.55035^{6}^
		$\hat{\theta }$	0.05303^{6}^	0.04833^{5}^	0.05363^{7}^	0.0393^{1}^	0.04658^{3}^	0.04628^{2}^	0.04818^{4}^
		$\hat{b}$	0.17757^{4}^	0.16806^{2}^	0.17771^{5}^	0.15295^{1}^	0.19089^{7}^	0.17713^{3}^	0.1901^{6}^
	AMREs	$\hat{\alpha }$	0.33947^{7}^	0.31671^{2}^	0.32111^{3}^	0.14019^{1}^	0.32514^{4}^	0.32912^{6}^	0.32803^{5}^
		$\hat{\theta }$	0.11971^{7}^	0.1144^{5}^	0.11877^{6}^	0.10476^{1}^	0.11245^{3}^	0.11169^{2}^	0.11415^{4}^
		$\hat{b}$	0.11355^{4}^	0.11012^{2}^	0.11329^{3}^	0.10648^{1}^	0.11821^{7}^	0.1138^{5}^	0.11652^{6}^
	∑ *Ranks*		53^{7}^	27^{2}^	39^{4}^	9^{1}^	43^{5}^	35^{3}^	46^{6}^
250	AESTs	$\hat{\alpha }$	1.75965	1.74781	1.76664	1.97747	1.76779	1.75287	1.74872
		$\hat{\theta }$	1.54285	1.53493	1.52976	1.47919	1.52756	1.52886	1.52667
		$\hat{b}$	2.96164	2.95796	2.94284	2.9365	2.9482	2.93604	2.94619
	ABs	$\hat{\alpha }$	0.46788^{5}^	0.47407^{7}^	0.45766^{2}^	0.08349^{1}^	0.4703^{6}^	0.46488^{4}^	0.46193^{3}^
		$\hat{\theta }$	0.10296^{2}^	0.10706^{7}^	0.10647^{6}^	0.10065^{1}^	0.10473^{4}^	0.1057^{5}^	0.10308^{3}^
		$\hat{b}$	0.206^{2}^	0.21662^{4}^	0.22687^{7}^	0.19964^{1}^	0.22497^{6}^	0.22488^{5}^	0.21243^{3}^
	AMSEs	$\hat{\alpha }$	0.28557^{5}^	0.29101^{7}^	0.276^{2}^	0.03403^{1}^	0.28875^{6}^	0.28344^{4}^	0.28142^{3}^
		$\hat{\theta }$	0.01703^{2}^	0.01872^{7}^	0.01841^{6}^	0.01611^{1}^	0.01798^{4}^	0.01816^{5}^	0.0173^{3}^
		$\hat{b}$	0.06566^{2}^	0.07229^{4}^	0.07897^{7}^	0.06238^{1}^	0.07825^{6}^	0.07746^{5}^	0.06938^{3}^
	AMREs	$\hat{\alpha }$	0.23394^{5}^	0.23703^{7}^	0.22883^{2}^	0.04174^{1}^	0.23515^{6}^	0.23244^{4}^	0.23096^{3}^
		$\hat{\theta }$	0.06864^{2}^	0.07137^{7}^	0.07098^{6}^	0.0671^{1}^	0.06982^{4}^	0.07047^{5}^	0.06872^{3}^
		$\hat{b}$	0.06867^{2}^	0.07221^{4}^	0.07562^{7}^	0.06655^{1}^	0.07499^{6}^	0.07496^{5}^	0.07081^{3}^
	∑ *Ranks*		27^{2.5}^	54^{7}^	45^{5}^	9^{1}^	48^{6}^	42^{4}^	27^{2.5}^
1000	AESTs	$\hat{\alpha }$	1.92492	1.95518	1.93926	2.00214	1.93488	1.93369	1.93856
		$\hat{\theta }$	1.50934	1.50749	1.50967	1.49144	1.51131	1.50836	1.50783
		$\hat{b}$	2.98387	2.99138	2.99511	2.98189	2.99306	2.98338	2.98694
	ABs	$\hat{\alpha }$	0.25047^{4}^	0.25209^{6}^	0.25438^{7}^	0.01791^{1}^	0.2508^{5}^	0.24445^{2}^	0.2479^{3}^
		$\hat{\theta }$	0.05175^{3}^	0.05278^{4}^	0.05325^{5}^	0.05095^{2}^	0.05334^{6}^	0.05594^{7}^	0.05091^{1}^
		$\hat{b}$	0.10296^{3}^	0.10321^{4}^	0.10838^{6}^	0.09467^{1}^	0.10175^{2}^	0.1124^{7}^	0.10361^{5}^
	AMSEs	$\hat{\alpha }$	0.0839^{6}^	0.08363^{4}^	0.08522^{7}^	0.00072^{1}^	0.08372^{5}^	0.08005^{2}^	0.08253^{3}^
		$\hat{\theta }$	0.00433^{3}^	0.00445^{4}^	0.00464^{6}^	0.00395^{1}^	0.00451^{5}^	0.00504^{7}^	0.00413^{2}^
		$\hat{b}$	0.01682^{2}^	0.01696^{5}^	0.01909^{6}^	0.01457^{1}^	0.01686^{3}^	0.02014^{7}^	0.01692^{4}^
	AMREs	$\hat{\alpha }$	0.12524^{4}^	0.12604^{6}^	0.12719^{7}^	0.00896^{1}^	0.1254^{5}^	0.12222^{2}^	0.12395^{3}^
		$\hat{\theta }$	0.0345^{3}^	0.03519^{4}^	0.0355^{5}^	0.03397^{2}^	0.03556^{6}^	0.03729^{7}^	0.03394^{1}^
		$\hat{b}$	0.03432^{3}^	0.0344^{4}^	0.03613^{6}^	0.03156^{1}^	0.03392^{2}^	0.03747^{7}^	0.03454^{5}^
	∑ *Ranks*		31^{3}^	41^{5}^	55^{7}^	11^{1}^	39^{4}^	48^{6}^	27^{2}^

**Table 3:** Simulation results of the AESTs, ABs, AMSEs, and AMREs of ExMED for (*α* = 2.5, *θ* = 0.75, *b* = 2).

n	Est.	Est. Par.	MLE	ADE	CVME	MPSE	LSE	RTADE	WLSE
20	AESTs	$\hat{\alpha }$	1.40439	1.36057	1.46854	1.80795	1.4303	1.37227	1.39436
		$\hat{\theta }$	0.86295	0.86284	0.84003	0.78899	0.84056	0.86913	0.83761
		$\hat{b}$	1.89359	1.87939	1.85802	1.80456	1.85293	1.87194	1.80552
	ABs	$\hat{\alpha }$	1.33594^{4}^	1.37185^{6}^	1.23695^{2}^	0.86488^{1}^	1.27424^{3}^	1.38149^{7}^	1.34999^{5}^
		$\hat{\theta }$	0.1829^{5}^	0.18482^{6}^	0.17262^{3}^	0.16996^{2}^	0.17571^{4}^	0.18784^{7}^	0.16944^{1}^
		$\hat{b}$	0.50686^{1}^	0.54325^{3}^	0.55954^{5}^	0.56912^{6}^	0.58912^{7}^	0.5317^{2}^	0.54495^{4}^
	AMSEs	$\hat{\alpha }$	2.16439^{4}^	2.259^{6}^	2.05663^{2}^	1.42915^{1}^	2.10069^{3}^	2.31154^{7}^	2.22224^{5}^
		$\hat{\theta }$	0.05471^{5}^	0.05666^{6}^	0.05088^{3}^	0.04532^{1}^	0.0513^{4}^	0.05879^{7}^	0.04798^{2}^
		$\hat{b}$	0.3791^{1}^	0.43486^{4}^	0.45304^{5}^	0.45317^{6}^	0.50484^{7}^	0.40851^{2}^	0.43212^{3}^
	AMREs	$\hat{\alpha }$	0.53438^{4}^	0.54874^{6}^	0.49478^{2}^	0.34595^{1}^	0.50969^{3}^	0.5526^{7}^	0.54^{5}^
		$\hat{\theta }$	0.24387^{5}^	0.24643^{6}^	0.23016^{3}^	0.22661^{2}^	0.23428^{4}^	0.25045^{7}^	0.22591^{1}^
		$\hat{b}$	0.25343^{1}^	0.27163^{3}^	0.27977^{5}^	0.28456^{6}^	0.29456^{7}^	0.26585^{2}^	0.27248^{4}^
	∑ *Ranks*		30^{3}^	46^{6}^	30^{3}^	26^{1}^	42^{5}^	48^{7}^	30^{3}^
70	AESTs	$\hat{\alpha }$	1.76706	1.70669	1.71125	2.24085	1.70763	1.66429	1.65545
		$\hat{\theta }$	0.81519	0.8088	0.80994	0.75637	0.80287	0.81537	0.80721
		$\hat{b}$	1.92156	1.89562	1.93266	1.89134	1.87381	1.89053	1.86246
	ABs	$\hat{\alpha }$	1.0871^{3}^	1.0854^{2}^	1.10538^{5}^	0.38575^{1}^	1.10336^{4}^	1.11998^{7}^	1.11338^{6}^
		$\hat{\theta }$	0.10588^{6}^	0.10362^{3}^	0.1041^{5}^	0.09547^{1}^	0.10365^{4}^	0.11049^{7}^	0.10258^{2}^
		$\hat{b}$	0.32294^{2}^	0.33201^{4}^	0.35839^{7}^	0.31333^{1}^	0.3448^{5}^	0.34902^{6}^	0.33102^{3}^
	AMSEs	$\hat{\alpha }$	1.48828^{3}^	1.47849^{2}^	1.54149^{5}^	0.50952^{1}^	1.52968^{4}^	1.56996^{7}^	1.55018^{6}^
		$\hat{\theta }$	0.0188^{6}^	0.01809^{3}^	0.01832^{4}^	0.01553^{1}^	0.01864^{5}^	0.02067^{7}^	0.01767^{2}^
		$\hat{b}$	0.16323^{2}^	0.1676^{4}^	0.20296^{7}^	0.14576^{1}^	0.17885^{5}^	0.18046^{6}^	0.16381^{3}^
	AMREs	$\hat{\alpha }$	0.43484^{3}^	0.43416^{2}^	0.44215^{5}^	0.1543^{1}^	0.44134^{4}^	0.44799^{7}^	0.44535^{6}^
		$\hat{\theta }$	0.14117^{6}^	0.13817^{3}^	0.1388^{5}^	0.1273^{1}^	0.13819^{4}^	0.14732^{7}^	0.13677^{2}^
		$\hat{b}$	0.16147^{2}^	0.166^{4}^	0.1792^{7}^	0.15667^{1}^	0.1724^{5}^	0.17451^{6}^	0.16551^{3}^
	∑ *Ranks*		33^{3.5}^	27^{2}^	50^{6}^	9^{1}^	40^{5}^	60^{7}^	33^{3.5}^
100	AESTs	$\hat{\alpha }$	1.97887	1.93329	1.92976	2.36399	1.95779	1.90886	1.91754
		$\hat{\theta }$	0.78847	0.7896	0.79065	0.7402	0.78459	0.78678	0.78527
		$\hat{b}$	1.95597	1.94382	1.95762	1.90364	1.95332	1.94427	1.93899
	ABs	$\hat{\alpha }$	0.81213^{4}^	0.7995^{3}^	0.81813^{6}^	0.22693^{1}^	0.79776^{2}^	0.81756^{5}^	0.82039^{7}^
		$\hat{\theta }$	0.07959^{3}^	0.07989^{5}^	0.08105^{7}^	0.0737^{1}^	0.07983^{4}^	0.07814^{2}^	0.08054^{6}^
		$\hat{b}$	0.24952^{1}^	0.25558^{3}^	0.27564^{6}^	0.26169^{4}^	0.28135^{7}^	0.25119^{2}^	0.27351^{5}^
	AMSEs	$\hat{\alpha }$	0.84182^{4}^	0.83011^{2}^	0.87608^{7}^	0.2294^{1}^	0.83148^{3}^	0.87404^{6}^	0.86103^{5}^
		$\hat{\theta }$	0.0103^{3}^	0.01087^{5}^	0.01103^{7}^	0.00818^{1}^	0.01069^{4}^	0.01006^{2}^	0.01092^{6}^
		$\hat{b}$	0.09607^{1}^	0.09903^{3}^	0.11422^{6}^	0.10122^{4}^	0.11969^{7}^	0.09643^{2}^	0.11168^{5}^
	AMREs	$\hat{\alpha }$	0.32485^{4}^	0.3198^{3}^	0.32725^{6}^	0.09077^{1}^	0.3191^{2}^	0.32702^{5}^	0.32816^{7}^
		$\hat{\theta }$	0.10612^{3}^	0.10652^{5}^	0.10807^{7}^	0.09827^{1}^	0.10644^{4}^	0.10419^{2}^	0.10739^{6}^
		$\hat{b}$	0.12476^{1}^	0.12779^{3}^	0.13782^{6}^	0.13084^{4}^	0.14068^{7}^	0.12559^{2}^	0.13675^{5}^
	∑ *Ranks*		24^{2}^	32^{4}^	58^{7}^	18^{1}^	40^{5}^	28^{3}^	52^{6}^
250	AESTs	$\hat{\alpha }$	2.22736	2.21742	2.19466	2.48545	2.17682	2.23236	2.19208
		$\hat{\theta }$	0.76653	0.7675	0.76477	0.74176	0.76662	0.76763	0.7686
		$\hat{b}$	1.98	1.97584	1.97001	1.9625	1.96777	1.98284	1.98737
	ABs	$\hat{\alpha }$	0.5598^{2}^	0.56427^{4}^	0.56402^{3}^	0.04848^{1}^	0.58427^{7}^	0.56568^{5}^	0.58384^{6}^
		$\hat{\theta }$	0.04846^{2}^	0.05054^{7}^	0.04946^{4}^	0.04401^{1}^	0.04981^{5}^	0.05009^{6}^	0.0494^{3}^
		$\hat{b}$	0.16523^{2}^	0.16803^{5}^	0.1677^{3}^	0.15802^{1}^	0.17195^{7}^	0.16826^{6}^	0.16781^{4}^
	AMSEs	$\hat{\alpha }$	0.41346^{2}^	0.41908^{3}^	0.42886^{5}^	0.02554^{1}^	0.45095^{7}^	0.4241^{4}^	0.4458^{6}^
		$\hat{\theta }$	0.00387^{2}^	0.00413^{7}^	0.00396^{3}^	0.00315^{1}^	0.004^{4}^	0.00407^{5.5}^	0.00407^{5.5}^
		$\hat{b}$	0.04273^{2}^	0.044^{4}^	0.04452^{6}^	0.03879^{1}^	0.0457^{7}^	0.04403^{5}^	0.04325^{3}^
	AMREs	$\hat{\alpha }$	0.22392^{2}^	0.22571^{4}^	0.22561^{3}^	0.01939^{1}^	0.23371^{7}^	0.22627^{5}^	0.23353^{6}^
		$\hat{\theta }$	0.06461^{2}^	0.06738^{7}^	0.06594^{4}^	0.05868^{1}^	0.06642^{5}^	0.06679^{6}^	0.06586^{3}^
		$\hat{b}$	0.08262^{2}^	0.08402^{5}^	0.08385^{3}^	0.07901^{1}^	0.08597^{7}^	0.08413^{6}^	0.0839^{4}^
	∑ *Ranks*		18^{2}^	46^{5}^	34^{3}^	9^{1}^	56^{7}^	48.5^{6}^	40.5^{4}^
1000	AESTs	$\hat{\alpha }$	2.41132	2.40957	2.40182	2.50087	2.41739	2.41981	2.42248
		$\hat{\theta }$	0.75465	0.75578	0.75508	0.74502	0.75423	0.75438	0.75405
		$\hat{b}$	1.99371	1.99995	1.98942	1.98253	1.99467	1.99	1.99139
	ABs	$\hat{\alpha }$	0.30799^{2}^	0.31027^{3}^	0.31263^{4}^	0.00754^{1}^	0.31968^{6}^	0.3174^{5}^	0.3212^{7}^
		$\hat{\theta }$	0.02197^{1}^	0.02369^{5}^	0.02422^{7}^	0.02233^{2}^	0.02319^{3}^	0.02361^{4}^	0.0241^{6}^
		$\hat{b}$	0.08037^{2}^	0.08284^{4}^	0.08649^{6}^	0.07516^{1}^	0.08957^{7}^	0.08116^{3}^	0.08632^{5}^
	AMSEs	$\hat{\alpha }$	0.12891^{2}^	0.12958^{3}^	0.13016^{4}^	0.00012^{1}^	0.13624^{7}^	0.13491^{6}^	0.13424^{5}^
		$\hat{\theta }$	0.00077^{1}^	9*e* − 04^{5}^	0.00091^{6}^	0.00078^{2}^	0.00084^{3}^	0.00088^{4}^	0.00094^{7}^
		$\hat{b}$	0.01022^{2}^	0.01091^{4}^	0.01153^{5}^	0.01003^{1}^	0.01253^{7}^	0.01031^{3}^	0.01165^{6}^
	AMREs	$\hat{\alpha }$	0.1232^{2}^	0.12411^{3}^	0.12505^{4}^	0.00302^{1}^	0.12787^{6}^	0.12696^{5}^	0.12848^{7}^
		$\hat{\theta }$	0.02929^{1}^	0.03159^{5}^	0.0323^{7}^	0.02978^{2}^	0.03093^{3}^	0.03148^{4}^	0.03213^{6}^
		$\hat{b}$	0.04019^{2}^	0.04142^{4}^	0.04324^{6}^	0.03758^{1}^	0.04479^{7}^	0.04058^{3}^	0.04316^{5}^
	∑ *Ranks*		15^{2}^	38^{4}^	48^{5}^	12^{1}^	49^{6}^	37^{3}^	53^{7}^

**Table 4:** Simulation results of the AESTs, ABs, AMSEs, and AMREs of ExMED for (*α* = 3, *θ* = 2.5, *b* = 0.5).

n	Est.	Est. Par.	MLE	ADE	CVME	MPSE	LSE	RTADE	WLSE
20	AESTs	$\hat{\alpha }$	1.77995	1.70335	1.93945	2.50236	1.8844	1.68403	1.75572
		$\hat{\theta }$	2.81441	2.75596	2.7096	2.41191	2.7177	2.80397	2.72748
		$\hat{b}$	0.48222	0.47005	0.4713	0.45577	0.46801	0.46824	0.47157
	ABs	$\hat{\alpha }$	1.23579^{4}^	1.33172^{6}^	1.07613^{2}^	0.5145^{1}^	1.13172^{3}^	1.33323^{7}^	1.29292^{5}^
		$\hat{\theta }$	0.63891^{4}^	0.61558^{1}^	0.62294^{2}^	0.64396^{5}^	0.6581^{6}^	0.66275^{7}^	0.62836^{3}^
		$\hat{b}$	0.09604^{1}^	0.10026^{2}^	0.10129^{3}^	0.10543^{5.5}^	0.10543^{5.5}^	0.1017^{4}^	0.106^{7}^
	AMSEs	$\hat{\alpha }$	2.62697^{4}^	2.83811^{6}^	2.30482^{2}^	1.06593^{1}^	2.42968^{3}^	2.89578^{7}^	2.71161^{5}^
		$\hat{\theta }$	0.66387^{5}^	0.62274^{4}^	0.62118^{3}^	0.61326^{1}^	0.68936^{6}^	0.69989^{7}^	0.61939^{2}^
		$\hat{b}$	0.01415^{1}^	0.01529^{3}^	0.01522^{2}^	0.016^{5}^	0.0168^{6}^	0.01542^{4}^	0.01684^{7}^
	AMREs	$\hat{\alpha }$	0.41193^{4}^	0.44391^{6}^	0.35871^{2}^	0.1715^{1}^	0.37724^{3}^	0.44441^{7}^	0.43097^{5}^
		$\hat{\theta }$	0.25556^{4}^	0.24623^{1}^	0.24917^{2}^	0.25758^{5}^	0.26324^{6}^	0.2651^{7}^	0.25134^{3}^
		$\hat{b}$	0.19209^{1}^	0.20052^{2}^	0.20258^{3}^	0.21086^{5.5}^	0.21086^{5.5}^	0.2034^{4}^	0.212^{7}^
	∑ *Ranks*		28^{2}^	31^{4}^	21^{1}^	30^{3}^	44^{5.5}^	54^{7}^	44^{5.5}^
70	AESTs	$\hat{\alpha }$	1.94976	1.96562	2.02165	2.88393	1.99739	1.89607	1.94085
		$\hat{\theta }$	2.68027	2.63498	2.6795	2.39994	2.65307	2.68783	2.62896
		$\hat{b}$	0.48439	0.47851	0.48576	0.47629	0.48056	0.48218	0.473
	ABs	$\hat{\alpha }$	1.12036^{4}^	1.1533^{5}^	1.05112^{3}^	0.1347^{1}^	1.0421^{2}^	1.20716^{7}^	1.1963^{6}^
		$\hat{\theta }$	0.38491^{4}^	0.38382^{3}^	0.40652^{6}^	0.36629^{1}^	0.40386^{5}^	0.40762^{7}^	0.38041^{2}^
		$\hat{b}$	0.05991^{3}^	0.0602^{4}^	0.06044^{5}^	0.05541^{1}^	0.06305^{6}^	0.05903^{2}^	0.06354^{7}^
	AMSEs	$\hat{\alpha }$	2.02148^{4}^	2.03292^{5}^	1.8909^{3}^	0.21107^{1}^	1.87899^{2}^	2.18143^{7}^	2.12315^{6}^
		$\hat{\theta }$	0.26126^{4}^	0.24347^{2}^	0.27902^{6}^	0.21239^{1}^	0.27187^{5}^	0.28471^{7}^	0.25308^{3}^
		$\hat{b}$	0.00533^{3}^	0.0057^{4}^	0.00577^{5}^	0.0046^{1}^	0.00609^{6}^	0.0053^{2}^	0.00614^{7}^
	AMREs	$\hat{\alpha }$	0.37345^{4}^	0.38443^{5}^	0.35037^{3}^	0.0449^{1}^	0.34737^{2}^	0.40239^{7}^	0.39877^{6}^
		$\hat{\theta }$	0.15396^{4}^	0.15353^{3}^	0.16261^{6}^	0.14651^{1}^	0.16154^{5}^	0.16305^{7}^	0.15216^{2}^
		$\hat{b}$	0.11981^{3}^	0.12039^{4}^	0.12088^{5}^	0.11082^{1}^	0.1261^{6}^	0.11805^{2}^	0.12708^{7}^
	∑ *Ranks*		33^{2}^	35^{3}^	42^{5}^	9^{1}^	39^{4}^	48^{7}^	46^{6}^
100	AESTs	$\hat{\alpha }$	2.34048	2.30603	2.31868	2.95712	2.36434	2.39251	2.37004
		$\hat{\theta }$	2.58513	2.59713	2.6078	2.41076	2.58879	2.58387	2.57813
		$\hat{b}$	0.4927	0.49057	0.49395	0.4836	0.49107	0.49346	0.49077
	ABs	$\hat{\alpha }$	0.71791^{4}^	0.79225^{7}^	0.7311^{6}^	0.05628^{1}^	0.70144^{3}^	0.68885^{2}^	0.72343^{5}^
		$\hat{\theta }$	0.30737^{4}^	0.30892^{5}^	0.31762^{7}^	0.30111^{1}^	0.31701^{6}^	0.30254^{2}^	0.30451^{3}^
		$\hat{b}$	0.0455^{1}^	0.04961^{7}^	0.04915^{6}^	0.04787^{5}^	0.0474^{4}^	0.04714^{3}^	0.04618^{2}^
	AMSEs	$\hat{\alpha }$	1.00948^{5}^	1.09848^{7}^	1.02663^{6}^	0.05789^{1}^	0.99539^{4}^	0.9625^{2}^	0.99084^{3}^
		$\hat{\theta }$	0.15168^{4}^	0.15671^{5}^	0.16503^{7}^	0.13845^{1}^	0.16196^{6}^	0.14997^{3}^	0.1499^{2}^
		$\hat{b}$	0.00323^{1}^	0.00383^{6.5}^	0.00383^{6.5}^	0.00349^{5}^	0.00344^{3}^	0.00348^{4}^	0.00333^{2}^
	AMREs	$\hat{\alpha }$	0.2393^{4}^	0.26408^{7}^	0.2437^{6}^	0.01876^{1}^	0.23381^{3}^	0.22962^{2}^	0.24114^{5}^
		$\hat{\theta }$	0.12295^{4}^	0.12357^{5}^	0.12705^{7}^	0.12044^{1}^	0.1268^{6}^	0.12101^{2}^	0.1218^{3}^
		$\hat{b}$	0.09101^{1}^	0.09922^{7}^	0.0983^{6}^	0.09575^{5}^	0.09481^{4}^	0.09428^{3}^	0.09237^{2}^
	∑ *Ranks*		28^{4}^	56.5^{6}^	57.5^{7}^	21^{1}^	39^{5}^	23^{2}^	27^{3}^
250	AESTs	$\hat{\alpha }$	2.59867	2.62395	2.62979	2.99359	2.59509	2.6372	2.60287
		$\hat{\theta }$	2.54748	2.54851	2.55965	2.4454	2.53112	2.54047	2.5302
		$\hat{b}$	0.49544	0.49507	0.49792	0.49075	0.49297	0.49544	0.4931
	ABs	$\hat{\alpha }$	0.51672^{2}^	0.55558^{6}^	0.51893^{3}^	0.01475^{1}^	0.52534^{4}^	0.52795^{5}^	0.58341^{7}^
		$\hat{\theta }$	0.20269^{6}^	0.19189^{2}^	0.20274^{7}^	0.18855^{1}^	0.19417^{5}^	0.19284^{4}^	0.19205^{3}^
		$\hat{b}$	0.03041^{6}^	0.03008^{4}^	0.02892^{2}^	0.02935^{3}^	0.03067^{7}^	0.02884^{1}^	0.03023^{5}^
	AMSEs	$\hat{\alpha }$	0.51678^{3}^	0.55169^{6}^	0.51232^{2}^	0.00479^{1}^	0.54144^{5}^	0.52261^{4}^	0.59022^{7}^
		$\hat{\theta }$	0.06596^{6}^	0.06078^{4}^	0.06694^{7}^	0.05481^{1}^	0.06046^{3}^	0.06012^{2}^	0.06261^{5}^
		$\hat{b}$	0.00143^{4.5}^	0.00146^{7}^	0.00137^{3}^	0.00131^{1}^	0.00144^{6}^	0.00132^{2}^	0.00143^{4.5}^
	AMREs	$\hat{\alpha }$	0.17224^{2}^	0.18519^{6}^	0.17298^{3}^	0.00492^{1}^	0.17511^{4}^	0.17598^{5}^	0.19447^{7}^
		$\hat{\theta }$	0.08108^{6}^	0.07676^{2}^	0.0811^{7}^	0.07542^{1}^	0.07767^{5}^	0.07714^{4}^	0.07682^{3}^
		$\hat{b}$	0.06083^{6}^	0.06017^{4}^	0.05784^{2}^	0.05871^{3}^	0.06134^{7}^	0.05768^{1}^	0.06047^{5}^
	∑ *Ranks*		41.5^{5}^	41^{4}^	36^{3}^	13^{1}^	46^{6}^	28^{2}^	46.5^{7}^
1000	AESTs	$\hat{\alpha }$	2.87208	2.90253	2.89882	2.99922	2.90661	2.89494	2.88224
		$\hat{\theta }$	2.50589	2.51379	2.50986	2.48825	2.5129	2.50852	2.51288
		$\hat{b}$	0.49803	0.49889	0.49856	0.49834	0.49914	0.49812	0.49837
	ABs	$\hat{\alpha }$	0.31072^{4}^	0.31527^{6}^	0.30209^{3}^	0.0046^{1}^	0.29145^{2}^	0.31197^{5}^	0.32293^{7}^
		$\hat{\theta }$	0.09474^{2}^	0.09995^{6}^	0.09976^{5}^	0.09225^{1}^	0.10125^{7}^	0.09787^{4}^	0.09757^{3}^
		$\hat{b}$	0.01476^{2}^	0.01505^{4}^	0.01537^{5}^	0.01445^{1}^	0.01541^{6}^	0.0148^{3}^	0.01585^{7}^
	AMSEs	$\hat{\alpha }$	0.16813^{6}^	0.16801^{5}^	0.16258^{3}^	4*e* − 05^{1}^	0.15254^{2}^	0.16674^{4}^	0.17246^{7}^
		$\hat{\theta }$	0.01395^{1}^	0.01576^{5}^	0.01584^{6}^	0.01444^{2}^	0.01609^{7}^	0.01491^{3}^	0.01494^{4}^
		$\hat{b}$	0.00034^{2.5}^	0.00035^{4}^	0.00036^{5}^	0.00032^{1}^	0.00037^{6}^	0.00034^{2.5}^	0.00038^{7}^
	AMREs	$\hat{\alpha }$	0.10357^{4}^	0.10509^{6}^	0.1007^{3}^	0.00153^{1}^	0.09715^{2}^	0.10399^{5}^	0.10764^{7}^
		$\hat{\theta }$	0.0379^{2}^	0.03998^{6}^	0.0399^{5}^	0.0369^{1}^	0.0405^{7}^	0.03915^{4}^	0.03903^{3}^
		$\hat{b}$	0.02953^{2}^	0.03011^{4}^	0.03073^{5}^	0.0289^{1}^	0.03081^{6}^	0.0296^{3}^	0.03169^{7}^
	∑ *Ranks*		24.5^{2}^	45^{6}^	39^{4}^	16^{1}^	44^{5}^	32.5^{3}^	51^{7}^

The following observations can be drawn from Tables 1–4.

- All the estimators reveal the consistency property, i.e., the MSE decreases when the sample size increases.
- ABs of all estimates decrease when *n* increases for all estimation methods.
- AMREs of all estimates decrease when *n* increases for all estimation methods.
- In terms of the performance of the estimation methods, we found that the MPSE estimates are the best estimators as they produce the least biases, and MSE with the least MRE for most of the configurations considered in our study. The next best estimators are the MLE estimates, followed by the ADE. The overall positions of the estimators are presented in Table 5, from which we can confirm the superiority of MPSE. In summary, based on Table 5, the performance ordering of estimators from best to worst for all parameter combinations is MPSE, MLE, ADE, WLSE, RTADE, CVME, and LSE.

**Table 5:** Partial and overall ranks of all the methods of estimation of ExMED by various values of *α*, *θ* and *b*.

Parameter	*n*	MLE	ADE	CVME	MPSE	LSE	RTADE	WLSE
*α* = 1.5, *θ* = 0.5, *b* = 0.75	20	1.5	7	1.5	4	6	5	3
	70	2	3	6.5	1	5	4	6.5
	100	2.5	6	7	1	5	4	2.5
	250	2	3	6	1	7	5	4
	1000	2	4	7	1	6	5	3
*α* = 2, *θ* = 1.5, *b* = 3	20	3	2	5	1	6	4	7
	70	2.5	4	7	1	5	6	2.5
	100	7	2	4	1	5	3	6
	250	2.5	7	5	1	6	4	2.5
	1000	3	5	7	1	4	6	2
*α* = 2.5, *θ* = 0.75, *b* = 2	20	3	6	3	1	5	7	3
	70	3.5	2	6	1	5	7	3.5
	100	2	4	7	1	5	3	6
	250	2	5	3	1	7	6	4
	1000	2	4	5	1	6	3	7
*α* = 3, *θ* = 2.5, *b* = 0.5	20	2	4	1	3	5.5	7	5.5
	70	2	3	5	1	4	7	6
	100	4	6	7	1	5	2	3
	250	5	4	3	1	6	2	7
	1000	2	6	4	1	5	3	7
∑ Ranks		55.5	87	100	25	108.5	93	91
Overall Rank		2	3	6	1	7	5	4

## 7 Data analysis

In this section, we use three real data sets from the fields of medicine and geology to explain the superiority of the proposed model in fitting these data sets over other related models. The first data was set about the remission times (in months) of a random sample of 128 bladder cancer patients, which was introduced in Lee and Wang [24]. The second data set consists of measurements made on patients with malignant melanoma. Each patient had their tumor removed by surgery at the Department of Plastic Surgery, University Hospital of Odense, Denmark, from 1962 to 1977. It consists of 7 variables, each with 205 observations; we studied the sixth variable (thickness: Tumour thickness in mm). It was obtained from Andersen et al. [25]. The third data set gives peak accelerations measured at various observation stations for 23 earthquakes in California and is referred to in [26] by Joyner and Boore. It consists of 5 variables and each variable consists of 182 observations, we studied the fourth variable [dist: numeric Station-hypocenter distance (km)]. The numerical values of data sets are given in Tables 6–8, respectively.

**Table 6:** Numerical values of the first data set.

14.76	6.54	2.75	7.87	6.93	10.34	3.25	5.06	0.4	12.63	5.32	12.07	5.41	23.63	1.26
15.96	4.33	5.32	34.26	9.02	3.31	11.64	25.74	7.28	20.28	0.81	5.49	4.98	2.09	4.18
19.13	4.26	3.36	5.71	3.64	3.48	2.02	43.01	8.37	17.14	2.26	2.62	5.85	5.17	6.94
13.29	5.62	7.63	9.74	7.09	5.41	36.66	7.32	46.12	3.36	4.87	14.77	4.4	32.15	22.69
1.46	7.66	10.06	0.51	79.05	6.25	6.76	0.5	2.46	3.57	4.51	18.1	3.7	0.2	1.4
4.5	4.34	17.36	5.09	2.54	9.22	8.26	7.39	21.73	10.75	0.9	14.24	1.05	2.02	7.62
2.69	7.59	3.52	1.35	2.83	11.25	13.8	8.66	12.02	25.82	16.62	11.79	1.76	26.31	2.64
2.07	9.47	7.93	8.65	3.82	17.12	6.97	14.83	2.23	3.02	11.98	12.03	10.66	13.11	7.26
8.53	4.23	1.19	2.69	5.34	3.88	2.87	0.08							

**Table 7:** Numerical values of the second data set.

6.76	0.65	1.34	2.9	12.08	4.84	5.16	3.22	12.88	7.41	4.19	0.16	3.87	4.84	2.42
12.56	5.8	7.06	5.48	7.73	13.85	2.34	4.19	4.04	4.84	0.32	8.54	2.58	3.56	3.54
0.97	4.83	1.62	6.44	14.66	2.58	3.87	3.54	1.34	2.24	3.87	3.54	17.42	1.29	3.22
1.29	4.51	8.38	1.94	0.16	2.58	1.29	0.16	1.62	1.29	2.1	0.32	0.81	1.13	5.16
1.62	1.37	0.24	0.81	1.29	1.29	0.97	1.13	5.8	1.29	0.48	1.62	2.26	0.58	0.97
2.58	0.81	3.54	0.97	1.78	1.94	1.29	3.22	1.53	1.29	1.62	1.62	0.32	4.84	1.29
0.97	3.06	3.54	1.62	2.58	1.94	0.81	7.73	0.97	12.88	2.58	4.09	0.64	0.97	3.22
1.62	3.87	0.32	0.32	3.22	2.26	3.06	2.58	0.65	1.13	0.81	0.97	1.76	1.94	0.65
0.97	5.64	9.66	0.1	5.48	2.26	4.83	0.97	0.97	5.16	0.81	2.9	3.87	1.94	0.16
0.64	2.26	1.45	4.82	1.29	7.89	0.81	3.54	1.29	0.64	3.22	1.45	0.48	1.94	0.16
0.16	1.29	1.94	3.54	0.81	0.65	7.09	0.16	1.62	1.62	1.29	6.12	0.48	0.64	3.22
1.94	2.58	2.58	0.81	0.81	3.22	0.32	3.22	2.74	4.84	1.62	0.65	1.45	0.65	1.29
1.62	3.54	3.22	0.65	1.03	7.09	1.29	0.65	1.78	12.24	8.06	0.81	2.1	3.87	0.65
1.94	0.65	2.1	1.94	1.13	7.06	6.12	0.48	2.26	2.9					

**Table 8:** Numerical values of the third data set.

12	148	42	85	107	109	156	224	293	359	370	8	16.1	63.6	6.6
9.3	13	17.3	105	112	123	105	122	141	200	45	130	147	187	197
203	211	62	62	19	21	13	22	29	17	19.6	20.2	21.1	21.9	24.2
66	87	23.4	24.6	25.7	28.6	37.4	46.7	56.9	60.7	61.4	62	64	82	88
91	31	45	145	300	5	50	16	17	8	10	10	8	32	30
31	2.9	3.2	7.6	25.4	32.9	92.2	1.2	1.6	9.1	3.7	5.3	7.4	17.9	19.2
23.4	30	38.9	23.5	26	0.5	0.6	1.3	1.4	2.6	3.8	4	5.1	6.2	6.8
7.5	7.6	8.4	8.5	8.5	10.6	12.6	12.7	12.9	14	15	16	17.7	18	22
22	23	23.2	29	32	32.7	36	43.5	49	60	64	7.5	8.8	8.9	9.4
9.7	9.7	10.5	10.5	12	12.2	12.8	14.6	14.9	17.6	23.9	25	10.8	15.7	16.7
20.8	28.5	33.1	40.3	4	10.1	11.1	17.7	22.5	26.5	29	30.9	37.8	48.3	5.8
12	12.1	20.5	20.5	25.3	35.9	36.1	36.3	38.5	41.4	43.6	44.4	46.1	47.1	47.7
49.2	53.1													

We compare the proposed distribution with some other well-known and related competing distributions, including Modi exponential distribution (MED) [14], modified kies exponential distribution (MKED) [27], alpha power exponential distribution (APED) [10], exponential distribution (ED), exponentiated exponential distribution (ExED), generalized log-logistic exponential distribution (GLLED) [28], linear exponential distribution (LNED) [29], logistic exponential distribution (LED) [30], Marshall Olkin exponential distribution (MOED) [3], Nadarajah Haghighi exponential distribution (NHED) [31], odd exponentiated half logistic exponential distribution (OExHLED) [32], odd inverse Pareto exponential distribution (OIPRED) [33], transmuted exponential distribution (TED) [29] and transmuted generalized exponential distribution (TGED) [34] distribution.

The comparison models can be compared using some discrimination measures such as the Akaike information criterion (AKIC), consistent Akaike information criterion (CAKIC), and Hannan–Quinn information (HAQUIC) criterion. Further discrimination measures include Anderson Darling (AD), Cramér–von Mises (CV), and Kolmogorov–Smirnov (KS) with its *p*-value (KSPV).

The MLEs and the analytical measures are computed using the Wolfram Mathematica version 12.0. Tables 9–14 give analytical measures along with the MLEs and their standard errors for the three data sets, respectively. The results in these tables indicate that the ExMED provides better fits than other competing models and could be chosen as an adequate model to analyze medicine (cancer) and geology (earthquakes) data sets.

**Table 9:** Discrimination measures of the ExMED model and other competing models for the first data set.

Model	-L	AIKC	CAKIC	HAQUIC	Estimates
ExMED	410.343	826.686	826.88	830.163	$\hat{\alpha }=0.282701\;\left(0.282701\right)$
					$\hat{\theta }=1.65111\;\left(0.210784\right)$
					$\hat{b}=0.0708053\;\left(0.0269109\right)$
MED	416.875	839.751	839.944	843.227	$\hat{\alpha }=0.412949\;\left(0.200264\right)$
					$\hat{\beta }=2.26687\times 10^{-26}\;\left(8.61185\times 10^{-14}\right)$
					$\hat{b}=0.0700735\;\left(0.00735464\right)$
ED	414.342	830.684	830.716	831.843	$\hat{\lambda }=0.106774\;\left(0.00943756\right)$
ExED	413.078	830.155	830.251	832.473	$\hat{\alpha }=0.121167\;\left(0.0135731\right)$
					$\hat{\lambda }=1.21795\;\left(0.148836\right)$
MKED	436.3	876.6	876.696	878.917	$\hat{\alpha }=0.68449\;\left(0.0484592\right)$
					$\hat{\lambda }=0.0505707\;\left(0.00391696\right)$
APED	414.318	832.636	832.732	834.954	$\hat{\alpha }=1.1739\;\left(0.843675\right)$
					$\hat{a}=0.111329\;\left(0.0226351\right)$
GLLED	412.625	829.251	829.347	831.568	$\hat{\alpha }=1.16343\;\left(0.0915071\right)$
					$\hat{\lambda }=0.100691\;\left(0.0084821\right)$
LNED	414.343	832.686	832.782	835.004	$\hat{\beta }=0.106765\;\left(0.0126644\right)$
					$\hat{\theta }=9.1263\times 10^{-7}\;\left(0.000901893\right)$
LED	412.625	829.251	829.347	831.568	$\hat{\alpha }=1.16343\;\left(0.0915071\right)$
					$\hat{\lambda }=0.100691\;\left(0.0084821\right)$
MOED	414.326	832.652	832.748	834.97	$\hat{\alpha }=1.0558\;\left(0.321669\right)$
					$\hat{a}=0.109863\;\left(0.019869\right)$
NHED	414.225	832.451	832.547	834.769	$\hat{\alpha }=0.922722\;\left(0.151589\right)$
					$\hat{\lambda }=0.121652\;\left(0.0344149\right)$
OExHLED	416.637	839.275	839.468	842.751	$\hat{\alpha }=0.952669\;\left(0.109129\right)$
					$\hat{\lambda }=3.85897\times 10^{-6}\;\left(0.0000108\right)$
					$\hat{\theta }=37291\;\left(105202\right)$
OIPRED	411.379	828.759	828.952	832.235	$\hat{\alpha }=1.70777\;\left(0.397763\right)$
					$\hat{\lambda }=0.0923213\;\left(0.0199409\right)$
					$\hat{\beta }=0.401327\;\left(0.202246\right)$
TED	414.232	832.463	832.559	834.781	$\hat{\beta }=0.120941\;\left(0.0246486\right)$
					$\hat{\lambda }=-0.252574\;\left(0.406875\right)$
TGED	410.947	827.893	828.087	831.37	$\hat{\alpha }=1.30249\;\left(0.148927\right)$
					$\hat{\lambda }=0.698079\;\left(0.230111\right)$
					$\hat{\theta }=0.0877731\;\left(0.0183056\right)$
WD	414.087	832.174	832.27	834.491	$\hat{\alpha }=1.04783\;\left(0.0675775\right)$
					$\hat{\beta }=9.5607\;\left(0.852901\right)$

**Table 10:** Discrimination measures of the ExMED model and other competing models for the first data set.

Model	AD	CV	KS	KSPV
ExMED	0.212017	0.0280561	0.0350813	0.997493
MED	2.62883	0.432786	0.12382	0.0394902
ED	1.17363	0.178809	0.0846343	0.318331
ExED	0.713912	0.127903	0.0725147	0.51132
MKED	7.65589	1.52346	0.192592	0.00015039
APED	1.08542	0.166313	0.0793338	0.396109
GLLED	0.627656	0.113062	0.0691031	0.573997
LNED	1.17354	0.178799	0.0846212	0.31851
LED	0.627656	0.113062	0.0691031	0.573997
MOED	1.11321	0.170092	0.0811329	0.368477
NHED	1.30354	0.199018	0.0919199	0.229607
OExHLED	1.3853	0.256709	0.0950481	0.197787
OIPRED	0.401122	0.0628855	0.0478403	0.931331
TED	0.92932	0.150413	0.0724567	0.512362
TGED	0.336895	0.0550819	0.0520973	0.877983
WD	0.957709	0.153703	0.0700169	0.5569655

**Table 11:** Discrimination measures of the ExMED model and other competing models for the second data set.

Model	-L	AIKC	CAKIC	HAQUIC	Estimates
ExMED	417.12	840.25	840.37	844.28	$\hat{\alpha }=0.3645\;\left(0.26013\right)$
					$\hat{\theta }=1.694\;\left(0.16867\right)$
					$\hat{b}=0.26035\;\left(0.06256\right)$
MED	424.66	855.33	855.45	859.36	$\hat{\alpha }=6.2519\;\left(15567\right)$
					$\hat{\beta }=10.742\;\left(15257\right)$
					$\hat{b}=0.34248\;\left(0.02392\right)$
ED	424.66	851.33	851.35	852.67	$\hat{\lambda }=0.34248\;\left(0.02392\right)$
ExED	421.35	846.7	846.76	849.38	$\hat{\alpha }=0.40277\;\left(0.035114\right)$
					$\hat{\lambda }=1.2913\;\left(0.1259\right)$
MKED	444.9	893.8	893.86	896.49	$\hat{\alpha }=0.75479\;\left(0.043157\right)$
					$\hat{\lambda }=0.17934\;\left(0.01064\right)$
APED	424.73	853.47	853.53	856.16	$\hat{\alpha }=1.3898\times 10^{-}12\;\left(3.1322\times 10^{-11}\right)$
					$\hat{a}=0.013029\;\left(0.011193\right)$
GLLED	421.33	846.67	846.73	849.36	$\hat{\alpha }=1.1799\;\left(0.072556\right)$
					$\hat{\lambda }=0.32085\;\left(0.021236\right)$
LNED	424.66	853.33	853.39	856.02	$\hat{\beta }=0.34248\;\left(0.033643\right)$
					$\hat{\theta }=8.0404\times 10^{-7}\;\left(0.0081025\right)$
LED	421.33	846.67	846.73	849.36	$\hat{\alpha }=1.1799\;\left(0.072556\right)$
					$\hat{\lambda }=0.32085\;\left(0.021236\right)$
MOED	424.47	852.94	853.00	855.63	$\hat{\alpha }=1.1596\;\left(0.27231\right)$
					$\hat{a}=0.36943\;\left(0.04982\right)$
NHED	424.65	853.29	853.29	855.98	$\hat{\alpha }=1.0292\;\left(0.15717\right)$
					$\hat{\lambda }=0.32724\;\left(0.081916\right)$
OExHLED	428.34	862.69	862.81	866.72	$\hat{\alpha }=0.9569\;\left(0.087082\right)$
					$\hat{\lambda }=0.0070577\;\left(0.0049232\right)$
					$\hat{\theta }=63.6700\;\left(45.921\right)$
OIPRED	417.16	840.32	840.44	844.35	$\hat{\alpha }=2.2129\;\left(0.52762\right)$
					$\hat{\lambda }=0.29071\;\left(0.049984\right)$
					$\hat{\beta }=0.28299\;\left(0.12900\right)$
TED	424.09	852.18	852.24	854.87	$\hat{\beta }=0.40298\;\left(0.052035\right)$
					$\hat{\lambda }=-0.34089\;\left(0.25882\right)$
TGED	419.09	844.18	844.29	848.21	$\hat{\alpha }=1.3831\;\left(0.12492\right)$
					$\hat{\lambda }=0.55398\;\left(0.22479\right)$
					$\hat{\theta }=0.32028\;\left(0.053504\right)$
WD	423.33	850.67	850.73	853.36	$\hat{\alpha }=1.0908\;\left(0.056721\right)$
					$\hat{\beta }=3.0268\;\left(0.20516\right)$

**Table 12:** Discrimination measures of the ExMED model and other competing models for the second data set.

Model	AD	CV	KS	KSPV
ExMED	0.36899	0.051703	0.044632	0.80878
MED	1.8385	0.24108	0.099269	0.035185
ED	1.8385	0.24108	0.09927	0.035183
ExED	1.2108	0.21064	0.081469	0.13155
MKED	7.5317	1.4343	0.15032	0.000189
APED	1.997	0.26433	0.10529	0.021233
GLLED	1.1126	0.1865	0.079447	0.1503
LNED	1.8385	0.24108	0.099268	0.035188
LED	1.1126	0.1865	0.079447	0.1503
MOED	1.6193	0.21493	0.089445	0.075239
NHED	1.7834	0.23404	0.096848	0.042746
OExHLED	2.5274	0.45097	0.10294	0.025942
OIPRED	0.41114	0.052949	0.048687	0.71607
TED	1.4465	0.21696	0.07394	0.21234
TGED	0.7177	0.11656	0.068852	0.28552
WD	1.4002	0.21433	0.072036	0.23785

**Table 13:** Discrimination measures of the ExMED model and other competing models for the third data set.

Model	-L	AIKC	CAKIC	HAQUIC	Estimates
ExMED	862.457	1730.91	1731.05	1734.81	$\hat{\alpha }=0.0820391\;\left(0.0628519\right)$
					$\hat{\theta }=1.50902\;\left(0.124455\right)$
					$\hat{b}=0.007869\;\left(0.003245\right)$
MED	870.091	1746.18	1746.32	1750.08	$\hat{\alpha }=0.313565\;\left(6430.92\right)$
					$\hat{\beta }=0.403866\;\left(7141.98\right)$
					$\hat{b}=0.012802\;\left(0.002712\right)$
ED	877.236	1756.47	1756.49	1757.77	$\hat{\lambda }=0.0219283\;\left(0.001625\right)$
ExED	876.782	1757.56	1757.63	1760.16	$\hat{\alpha }=0.91119\;\left(0.08958\right)$
					$\hat{\lambda }=0.020581\;\left(0.002109\right)$
MKED	905.603	1815.21	1815.27	1817.8	$\hat{\alpha }=0.599268\;\left(0.036294\right)$
					$\hat{\lambda }=0.010271\;\left(0.000727\right)$
APED	868.183	1740.37	1740.43	1742.96	$\hat{\alpha }=0.039245\;\left(0.053019\right)$
					$\hat{a}=0.009053\;\left(0.003462\right)$
GLLED	877.09	1758.18	1758.25	1760.78	$\hat{\alpha }=0.965531\;\left(0.063210\right)$
					$\hat{\lambda }=0.0221767\;\left(0.001748\right)$
LNED	877.252	1758.5	1758.57	1761.1	$\hat{\beta }=0.021923\;\left(0.002018\right)$
					$\hat{\theta }=1\times 10^{-7}\;\left(0.0000262\right)$
LED	877.09	1758.18	1758.25	1760.78	$\hat{\alpha }=0.965531\;\left(0.06321\right)$
					$\hat{\lambda }=0.022176\;\left(0.001748\right)$
MOED	870.091	1744.18	1744.25	1746.78	$\hat{\alpha }=0.384999\;\left(0.110205\right)$
					$\hat{a}=0.012802\;\left(0.002712\right)$
NHED	869.832	1743.66	1743.73	1746.26	$\hat{\alpha }=0.63164\;\left(0.069494\right)$
					$\hat{\lambda }=0.05086\;\left(0.011423\right)$
OExHLED	886.979	1779.96	1780.09	1783.85	$\hat{\alpha }=0.715004\;\left(0.066259\right)$
					$\hat{\lambda }=.0000186\;\left(0.00003\right)$
					$\hat{\theta }=1279.14\;\left(2063.17\right)$
OIPRED	863.622	1733.24	1733.38	1737.14	$\hat{\alpha }=1.99957\;\left(0.420656\right)$
					$\hat{\lambda }=0.010369\;\left(0.002664\right)$
					$\hat{\beta }=0.119273\;\left(0.054421\right)$
TED	871.131	1746.26	1746.33	1748.86	$\hat{\beta }=0.015588\;\left(0.001929\right)$
					$\hat{\lambda }=0.64156\;\left(0.14662\right)$
TGED	871.106	1748.21	1748.35	1752.11	$\hat{\alpha }=1.02037\;\left(0.090908\right)$
					$\hat{\lambda }=0.638969\;\left(0.144097\right)$
					$\hat{\theta }=0.015913\;\left(0.0024\right)$
WD	874.402	1752.8	1752.87	1755.4	$\hat{\alpha }=0.883609\;\left(0.047648\right)$
					$\hat{\beta }=42.4615\;\left(3.77666\right)$

**Table 14:** Discrimination measures of the ExMED model and other competing models for the third data set.

Model	AD	CV	KS	KSPV
ExMED	0.281167	0.032627	0.03920	0.94239
MED	2.20749	0.346997	0.095267	0.073496
ED	4.32173	0.861772	0.126781	0.005755
ExED	3.68938	0.706354	0.116922	0.013801
MKED	13.681	2.87427	0.229701	9.12301 × 10^−9^
APED	1.57445	0.22539	0.087113	0.126261
GLLED	4.04853	0.79944	0.123508	0.007754
LNED	4.3258	0.862566	0.126829	0.00573
LED	4.04853	0.79944	0.123508	0.007754
MOED	2.20749	0.346997	0.095267	0.073496
NHED	1.96461	0.30151	0.090463	0.101687
OExHLED	5.97062	1.16355	0.145868	0.000865
OIPRED	0.554154	0.087211	0.051026	0.730476
TED	2.08897	0.352847	0.085415	0.140458
TGED	2.12436	0.364958	0.087151	0.125953
WD	2.76564	0.462896	0.091082	0.097613

**Fig 4:**
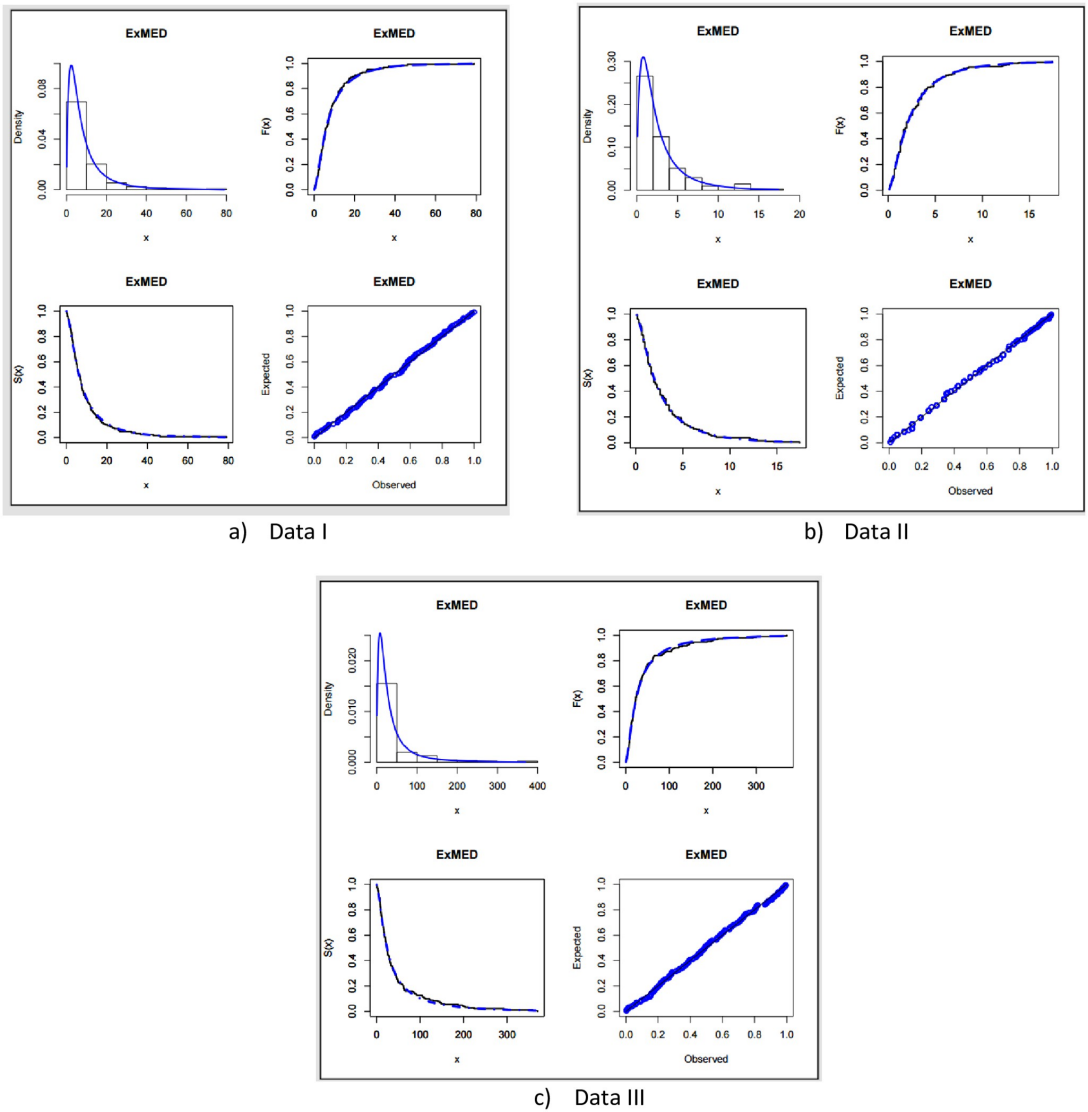
The estimated ExMED PDF, CDF, SF, and P–P plots.

**Fig 5:**
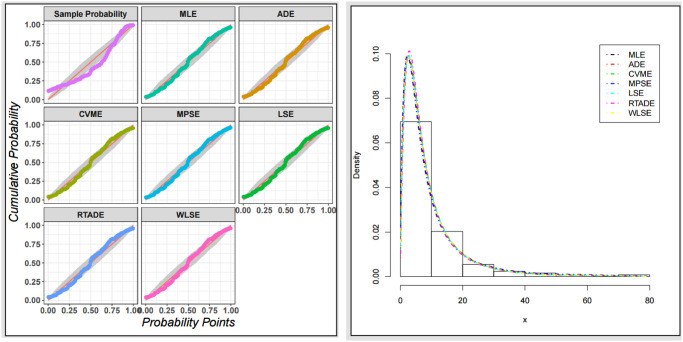
The P–P plots and histogram of data set I with the fitted ExMED PDFs for all estimation methods.

**Fig 6:**
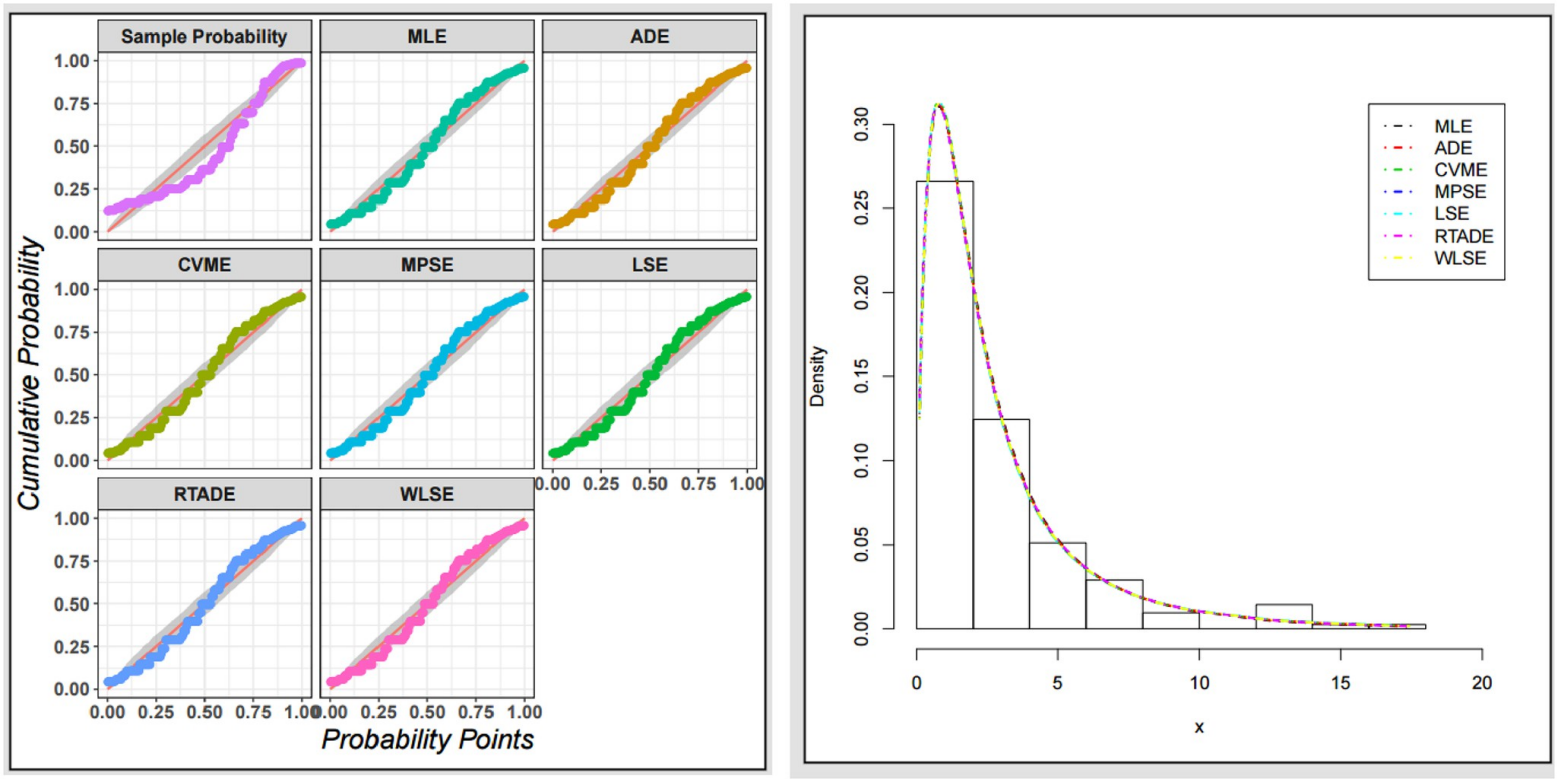
The P–P plots and histogram of data set II with the fitted ExMED PDFs for all estimation methods.

**Fig 7:**
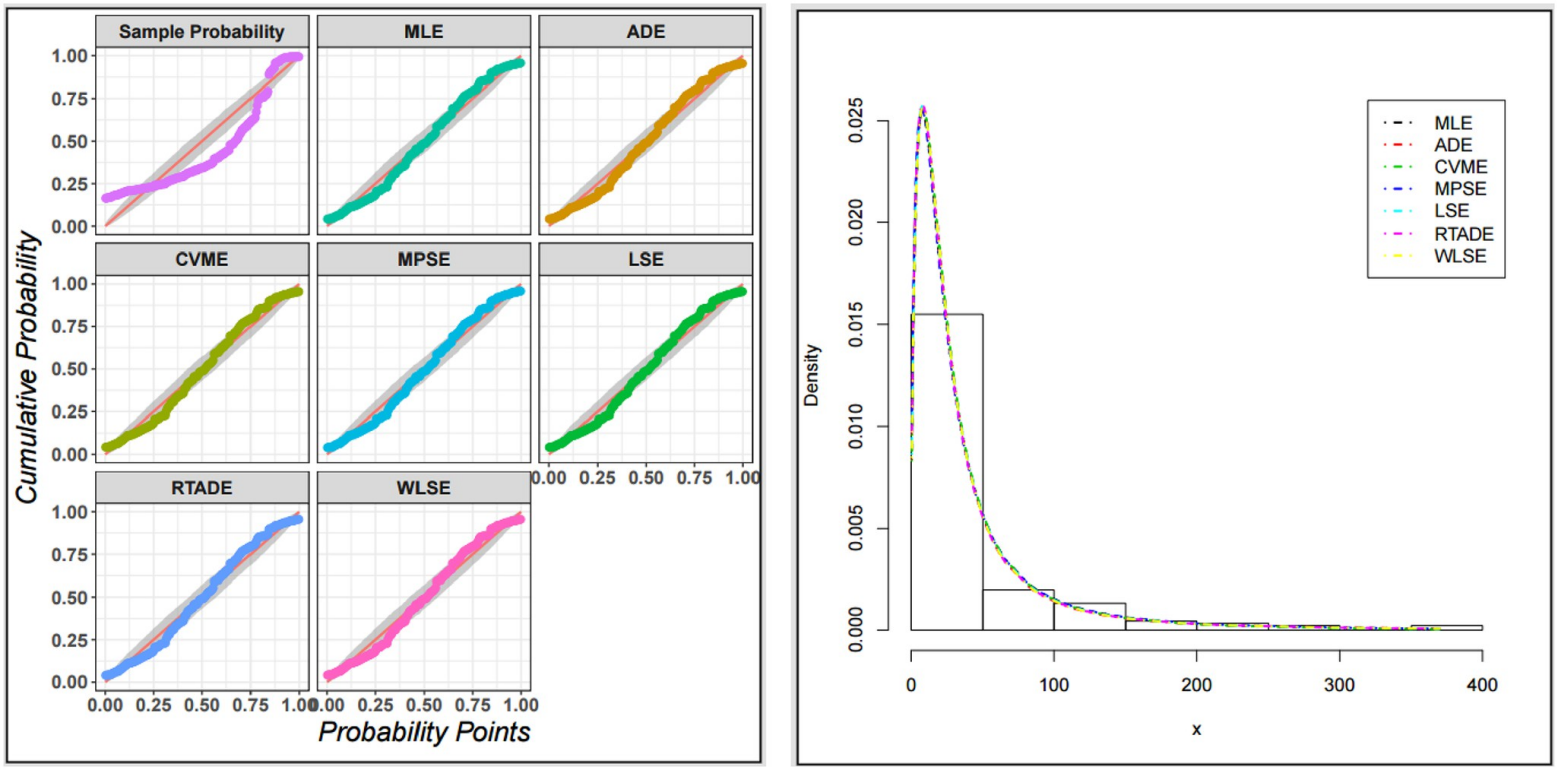
The P–P plots and histogram of data set III with the fitted ExMED PDFs for all estimation methods.

**Fig 8:**
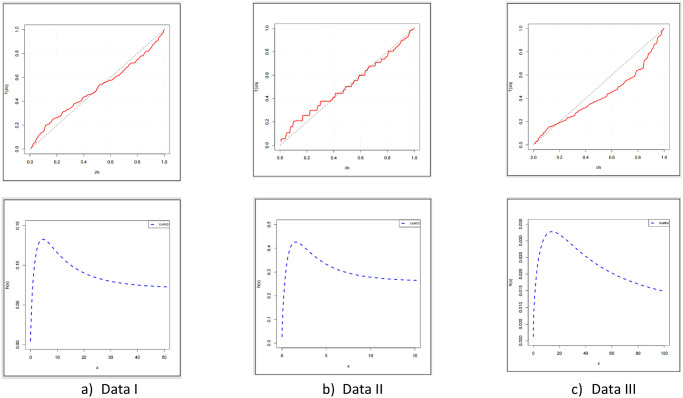
The TTT plots and estimated HRFs of ExMED.

**Fig 9:**
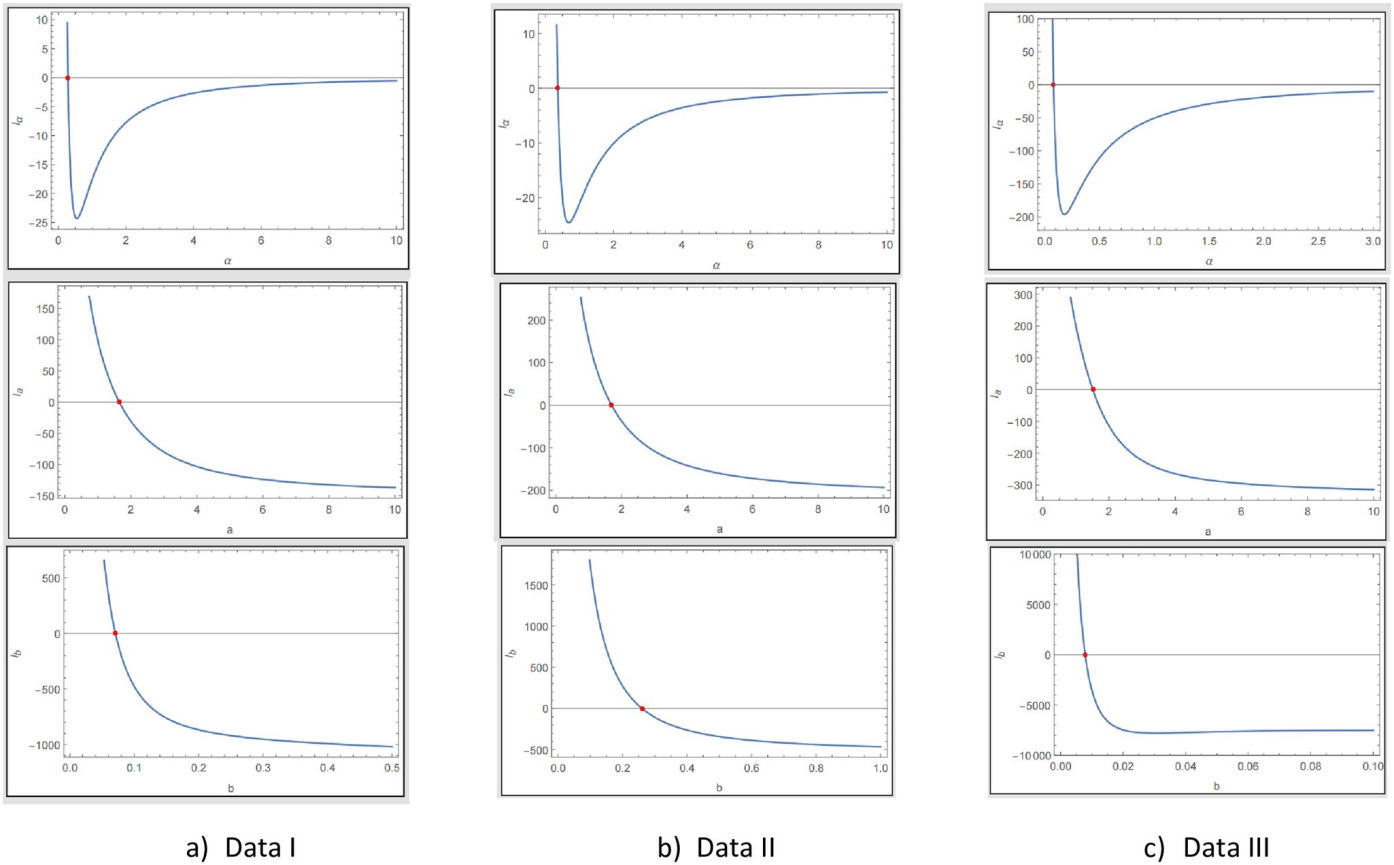
Existence and uniqueness of ExMED parameters.

**Fig 10:**
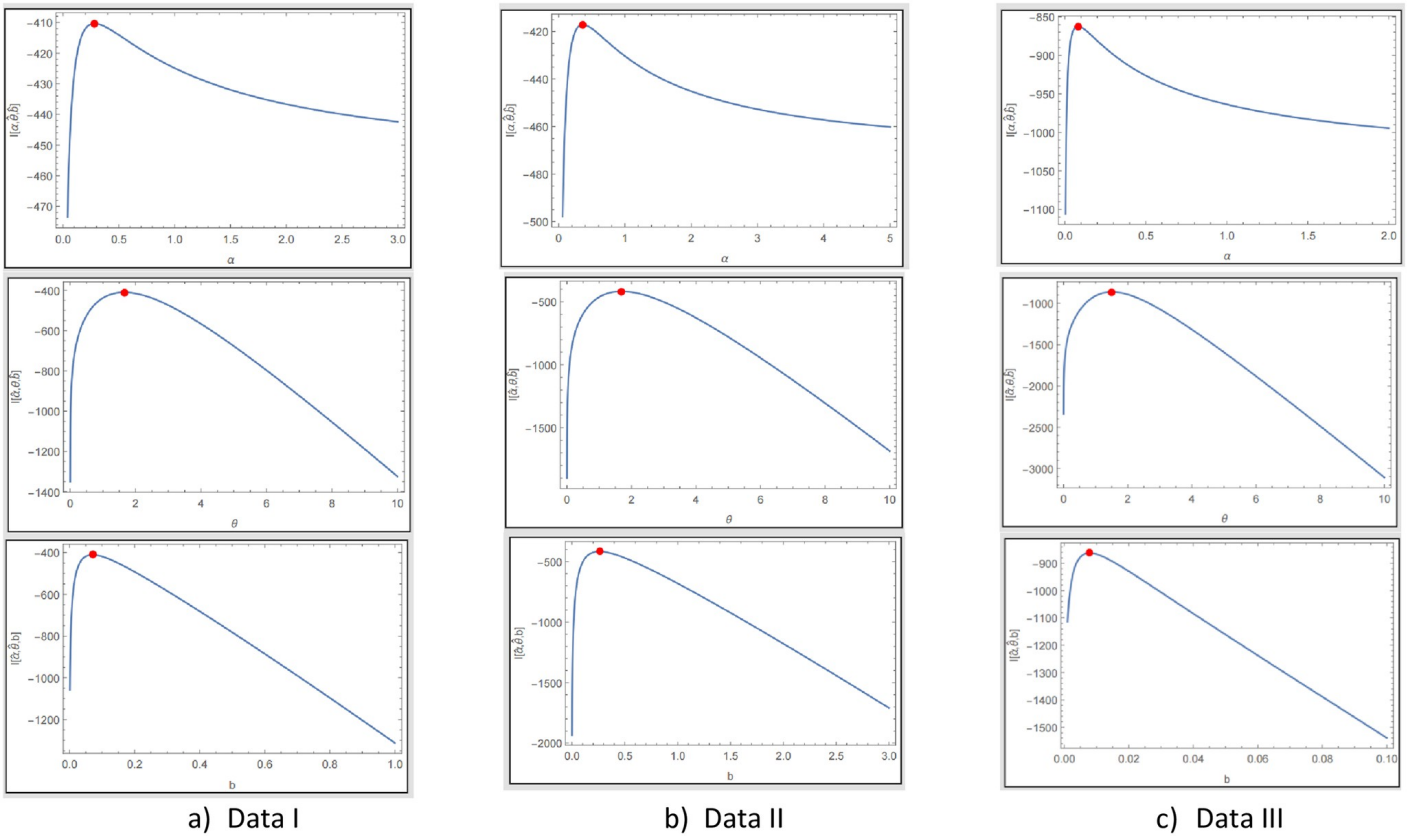
Curve of the log-likelihood function of ExMED.

The fitted PDF, CDF, SF, and probability-probability (P–P) plots of the ExMED for the three data sets are shown in Fig 4, respectively. Furthermore, we use the seven estimation approaches discussed in Section 4 to estimate the ExMED parameters. Table 15 reports the estimates of the ExMED parameters using these approaches and the numerical values of estimated parameters and negative log-likelihood along with goodness-of-fit for three data sets, respectively. Based on the values of KS and KSP listed in Table 15, we conclude that the seven estimation methods perform well in fitting the three data sets. The P–P plots and histogram of three data sets with the fitted ExMED density for various estimation methods are, respectively, shown in Figs 5–7 that support the results in Table 15. Fig 8 provides the TTT plots and plots of the estimates HRF of ExMED for the three data sets, respectively. They reveal that the ExME HRFs have unimodal shapes respectively. This fact agrees with the TTT plot based on each data set. The proposed model’s estimated parameters’ existence and uniqueness are shown graphically in Fig 9 for the three data sets. These estimated parameters were calculated using the NMaximize function in Wolfram Mathematica software version 12.0, which always attempts to find a global maximum of the objective function subject to the constraints given. Also, in Fig 10, we show that these estimated parameters maximize the log-likelihood function for the three data sets.

**Table 15:** The estimates and negative log-likelihood function of the ExMED along with goodness-of-fit measures for the three data sets.

Data	Method	$\hat{\alpha }$	$\hat{\theta }$	$\hat{\lambda }$	−L	AD	CV	KS	KSPV
Data I	MLE	0.282701	1.65111	0.0708053	410.343	0.212017	0.0280561	0.0350813	0.997493
	ADE	0.344222	1.72576	0.0817925	410.565	0.164938	0.0160885	0.0305205	0.999767
	CVME	0.3271145	1.80072	0.0840575	410.879	0.185769	0.0136377	0.031457	0.999585
	MPSE	0.169801	1.61755	0.0532711	410.642	0.342424	0.0466752	0.0403203	0.985372
	LSE	0.305352	1.77503	0.0801607	410.678	0.17155	0.0141608	0.0311388	0.999657
	RTADE	0.179622	1.86959	0.0670801	410.919	0.216178	0.0150543	0.0350307	0.997545
	WLSE	0.281907	1.74131	0.0756298	410.494	0.167492	0.0164161	0.0313968	0.9996
Data II	MLE	0.364498	1.69397	0.260355	417.124	0.368987	0.0517034	0.0446323	0.808776
	ADE	0.311066	1.6841	0.241144	417.181	0.359468	0.0502318	0.044517	0.811262
	CVME	0.259229	1.69812	0.224218	417.295	0.363011	0.0499603	0.0438941	0.824499
	MPSE	0.309992	1.66566	0.237403	417.295	0.363011	0.0499603	0.0438941	0.824499
	LSE	0.249866	1.68344	0.217767	417.38	0.369084	0.0502926	0.0454273	0.791362
	RTADE	0.330726	1.66822	0.245149	417.179	0.360589	0.0504437	0.0460054	0.778421
	WLSE	0.276411	1.6958	0.230177	417.245	0.361267	0.0500881	0.0435274	0.832131
Data III	MLE	0.0820391	1.50902	0.007869	862.457	0.281167	0.0326273	0.039206	0.942392
	ADE	0.0181121	1.53685	0.0031277	863.366	0.220735	0.0234106	0.0352372	0.977555
	CVME	0.089921	1.55099	0.0088572	862.58	0.327857	0.0301712	0.0378162	0.957066
	MPSE	0.0641536	1.47798	0.00646127	862.60	0.277137	0.0361253	0.0433513	0.883696
	LSE	0.0699648	1.5368	0.0074632	862.492	0.274399	0.0280116	0.0375738	0.959361
	RTADE	0.0271726	1.50846	0.003903	863.134	0.235143	0.0267686	0.0405061	0.92633
	WLSE	0.0255388	1.53393	0.00389001	863.126	0.221998	0.0242089	0.0360051	0.972343

## 8 Conclusion

A new family of life distributions called the extended Modi-G family is presented, and general expressions for some mathematical statistics properties of the new family, including quantile function, moments, moment generating function, incomplete moments, inequality curves, Rényi entropy, and Shannon entropy are derived. The maximum likelihood, ordinary least square, weighted least square, Anderson Darling, right-tailed Anderson-Darling, Cramér-von Mises, and maximum product of spacing methods were discussed to estimate the model parameters. A special sub-model called extended Modi exponential distribution was derived. Its density function can be symmetric, left-skewed, right-skewed, increasing, J-shape, and inverse J-shape along with upside-down bathtub, decreasing, decreasing-constant, increasing, and increasing constant hazard rate functions. Different data sets were analyzed, and the superiority of the extended Modi exponential distribution for fitting data sets was illustrated over other compared distributions.
